# Supplementary material for: Distinct dissociation rates of murine and human norovirus P-domain dimers suggest a role of dimer stability in virus-host interactions
Source: Commun Biol. 2022 Jun 9;5:563. doi: 10.1038/s42003-022-03497-4 (PMC9184547; doi:10.1038/s42003-022-03497-4)
Supplement: Supplementary file 2 — Supplementary Information [file 42003_2022_3497_MOESM2_ESM.pdf]

SUPPLEMENTARY INFORMATION

Distinct dissociation rates of murine and human norovirus P-domain dimers suggest a role of dimer stability in virus-host interactions

Robert Creutznacher[1,2], Thorben Maass[1], Jasmin Dülfer[3], Clara Feldmann[1], Veronika Hartmann[4], Miranda Sophie Lane[4], Jan Knickmann[4], Leon Torben Westermann[1], Lars Thiede[3,5], Thomas J. Smith[6], Charlotte Uetrecht[3,5,7], Alvaro Mallagaray[1], Christopher A. Waudby[8], Stefan Taube[4], and Thomas Peters[1]

Table of Contents

Supplementary Figures 3

Fig. S1: Comparison of structures of the MNV-1 P-dimer in the absence (left, PDB 3LQ6) and presence of GCDCA (right, PDB 6E47). 3

Fig. S2: Sequence alignment of MNV P-domains. 4

Fig. S3: pH-dependent stability of murine NoV P-domains. 5

Fig. S4: Calibration of analytical size exclusion chromatography column Superdex 75 Increase 3.2/300. 6

Fig. S5: SEC-MALS of MNV CW1 P-domains with different protein concentrations. 7

Fig. S6: The MNV P-domain has a weak tendency to form dimers. 8

Fig. S7: Assignment of $^{1}H,^{13}C$ cross peaks in a methyl-TROSY spectrum of MIL^{ProS}VP^{roS}A-labeled sample of MNV CW1 P-domain saturated with GCDCA. 10

Fig. S8: Methyl TROSY cross peaks reflect slow to fast-to-intermediate exchange upon titration of a sample of MIL^{ProS}V^{ProS}A-labeled P-domain of MNV CW1 with GCDCA. 11

Fig. S9: 2D line shape analysis of $^{1}H,^{13}C$ HMQC spectra of MIL^{proS}V^{proS}A-labeled MNV CW1 P-domain at increasing protein concentrations. 13

Fig. S10: Comparison of MNV and huNoV P-dimers (GII.4 Saga). 18

Fig. S11: Increase of thermostability of MNV P-domains and virions in the presence of GCDCA. 19

Fig. S12: Increase of MNV P-domain thermal stability by ligand binding is specific for GCDCA. 20

Fig. S13: Changes in NMR relaxation times indicate altered protein dynamics upon dimerization and ligand binding. 21

Fig. S14: MNV P-domain proteins titrated with GCDCA revert to their apo state during size exclusion chromatography (SEC). 22

Fig. S15: Overlay of sections of methyl TROSY spectra of MIL^{proS}V^{proS}A-labeled MNV CW1 P-domain in the presence and absence of GCDCA. 23

Fig. S16: Determination of the minimum threshold for significant chemical shift perturbations 24

Fig. S17: Histogram showing CSPs for the MNV CW1 P-domain upon addition of GCDCA. 25

Fig. S18: Comparison of GCDCA-induced changes to the MNV P-domain based on crystal structure analysis with NMR-derived chemical shift mapping. 26

Fig. S19: GCDCA but not TCA blocks neutralization of different monoclonal antibodies. 27

Fig. S20: GCDCA improves infectivity of MNV-1 and prevents neutralization with antibody A6.2 28

Fig. S21: Representative sections from $^{1}H,^{15}N$ TROSY HSQC spectra of MNV CW1 P-domain 29

Fig. S22: Full data set used for the TITAN analysis of GCDCA binding 30

Supplementary Tables 31

Tab. S1: Amino acid sequences of norovirus P-domain proteins studied. 31

Table S2: Dissociation constants and rate constants for dimerization of P-domains and binding of GCDCA to P-dimers. 32

Tab. S3: Transfer of assignments of ^{13}C-methyl groups for the GCDCA-bound form of MNV CW1 P-dimers to the apo-form. 33

Tab. S4: Additional acquisition parameters for 2D NMR experiments 35

Tab. S5: Final concentrations of precursors for MILVA-labeling of MNV-P-domains. 35

Table S6: PRODIGY server predictions for monomer-monomer interaction energies in P-domain dimers 36

Supplementary Notes 38

Supplementary Note 1: Shell scripts for NMR data processing with NMRPipe 38

Supplementary References 40

Supplementary Figures

Fig. S1: Comparison of structures of the MNV-1 P-dimer in the absence (left, PDB 3LQ6) and presence of GCDCA (right, PDB 6E47).

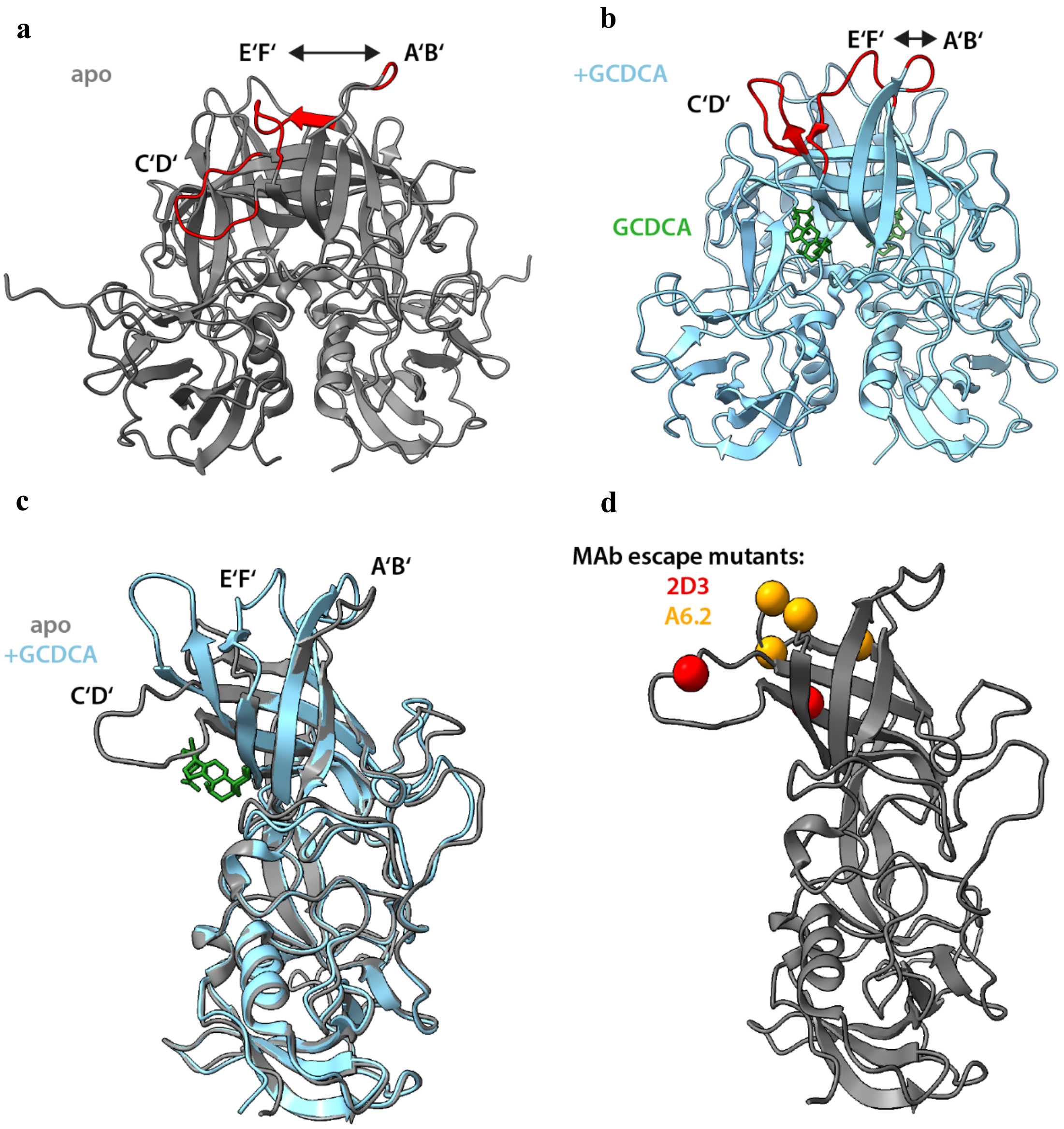

a MNV-1 P-dimer in the absence of GCDCA (PDB 3LQ6) with the C'D', E'F', and A'B' loops highlighted. The C'D' loop blocks access to the GCDCA binding pocket ("closed" conformation). **b** MNV-1 P-dimer in the presence of GCDCA (PDB 6E47). The C'D' loop has reoriented to allow GCDCA entering the binding pocket ("open" conformation). At the same time the E'F' and A'B' loops rearrange and come closer to each other. The distance between the loops in the open and closed form can be illustrated by measuring the distances between the Cα atoms in the two loops. For instance, the distance between Cα of Ala381 (E'F' loop) and Cα of Ser299 (A'B' loop) is 8 Å in the closed form and 37 Å in the open form. **c** Overlay of the open and closed P-domain conformations (only one monomer is shown) highlighting the loop reorientations. **d** Escape mutations for the monoclonal antibodies 2D3 and 4F9 (red, D348E, V339I) and A6.2 (yellow, A382K/R, D385E, V378F, L386F) are mapped on the open conformation of the MNV-1 P-domain.

Fig. S2: Sequence alignment of MNV P-domains.

```
CW1     ---------------GPRMVDLPVIQPRLCTHARWPAPVYGLLVDPSLPSNPQWQNGRVHVDGTLLGTTP 280
MNV07   -------------GPGSRMVDLPVLQPRLCTHARWPAPVYGLLVDPSLPSNPQWQNGRVHVDGTLLGTTP
CR10    -------------GPGSRMVDLPVLQPRLCTHARWPAPIYGLLVDPSLPSNPQWQNGRVHVDGTLLGTTP
                       *.*******:*************:*******************************

CW1     ISGSWVSCFAAEAAYEFQSGTGEVATFTLIEQDGSAYVPGDRAAPLGYPDFSGQLEIEVQTETTKTGDKL 350
MNV07   VSGSWVSCFAAEAAYEFQSGTGEVATFTLIEQDGSAYVPGDRAAPLGYPDFSGQLEIEVQTETTKAGDKL
CR10    VSGSWVSCFAAEAAYEFQSGTGEVATFTLIEQDGSAYVPGDRAAPLGYPDFSGQLEIEVQTETTKTGDKL
        :****************************************************************:****

CW1     KVTTFEMILGPTTNADQAPYQGRVFASVTAAASLDLVDGRVRAVPRSIYGFQDTIPEYNDGLLVPLAPPI 420
MNV07   KVTTFEMILGPTTNVDQAPYQGRVHASTSVTASLNLVDGRVRAVPRSIYSFQDVVPEYNDGLLVPLAPPI
CR10    KVTTFEMILGPTTNVDQAPYQGRVYASLTAVASLDLVDGRVRAVPRSIYGFQDVIPEYNDGLLVPLAPPI
        **************.*********.** :..***:**************.***.:***************

CW1     GPFLPGEVLLRFRTYMRQIDTADAAAEAIDCALPQEFVSWFASNAFTVQSEALLLRYRNTLTGQLLFECK 490
MNV07   GPFLPGEVLLRFRTYMRQIDSSDAAAEAIDCALPQEFISWFASNAFTVQSEALLLRYRNTLTGQLLFECK
CR10    GPFLPGEVLLRFRTYMRQLDTADAAAEAIDCALPQEFISWFASNAFTVQSDALLLRYRNTLTGQLLFECK
        ******************:*::***************:************:*******************

CW1     LYNEGYIALSYSGSGPLTFPTDGIFEVVSWVPRLYQLASV 530
MNV07   LYSEGYIALSYSGSGPLTFPTDGFFEVVSWVPRLYQLASV
CR10    LYSEGYIALSYSGSGPLTFPTDGFFEVVSWVPRLFQLASV
        **.********************:**********:*****
```

Recombinant CW1, MNV07 and CR10 P-domains from MNV have been used in this study. The sequences correspond to the GenBank entries given in Tab. S1. Multiple sequence alignment was done using the Clustal Omega algorithm[1-3]. ":" corresponds to conserved and "." to semi-conserved substitutions.

Fig. S3: pH-dependent stability of murine NoV P-domains.

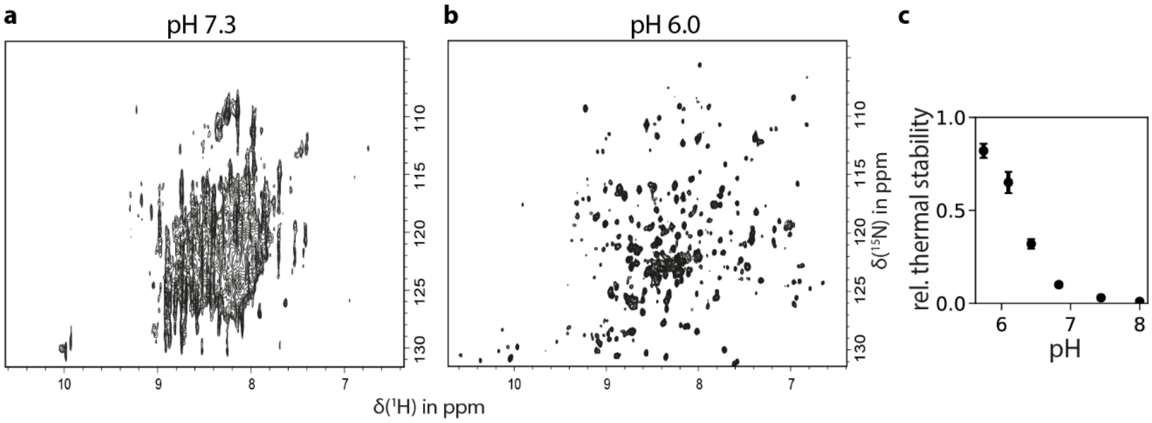

a Purification of MNV P-domains in buffers at pH 7.3 leads to irreversible protein unfolding and aggregation. A TROSY HSQC spectrum of [*U*-^{2}H,^{15}N] labeled MNV07 P-domains in 20 mM sodium phosphate buffer (pH* 7.3) shows little chemical shift dispersion and strong line broadening. **b** In contrast, samples in 20 mM sodium phosphate buffer, 100 mM NaCl (pH* 6) give spectra with dispersed, sharp NH signals indicating a well-ordered and stable protein. **c** The thermal stability of MNV P-domains decreases with increasing pH. P-domains from the strain CR10 were subjected to isothermal denaturation in 75 mM sodium phosphate buffer, 100 mM NaCl at different pH values at 45 °C and subsequent hydrophobic interaction chromatography (HIC). The UV absorption in HIC experiments can be used to quantify the amount of non-denatured protein. UV integrals were normalized against a non-heat-treated control. HIC experiments were performed as duplicates. The respective percentage of deviation is given as error bars. The spectra in **a** and **b** were acquired with 15 µM protein concentration and 672 scans, and 30 µM and 136 scans, respectively. Additionally, spectrum **a** was acquired with 128 increments in the indirect dimension. Other acquisition parameters are given in Tab. S4.

Fig. S4: Calibration of analytical size exclusion chromatography column Superdex 75 Increase 3.2/300.

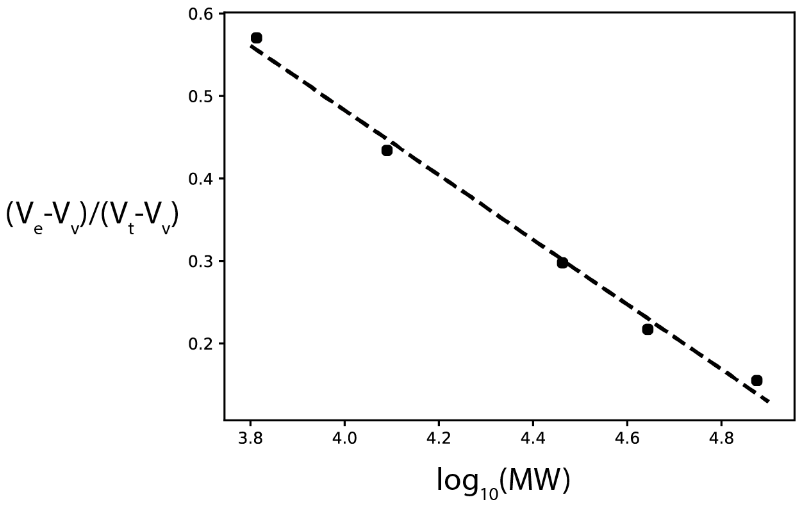

A mixture of five standard proteins with molecular weights 6.5 – 75 kDa (see methods) was applied to the column in 20 mM sodium acetate buffer, 100 mM NaCl, pH 5.3 at 4 °C. Linearized data (MW: molecular weight, V_e: observed elution volume, V_V: void volume, V_t: total column volume) yielded the calibration curve used for molecular weight estimates of P-domains in this study.

Fig. S5: SEC-MALS of MNV CW1 P-domains with different protein concentrations.

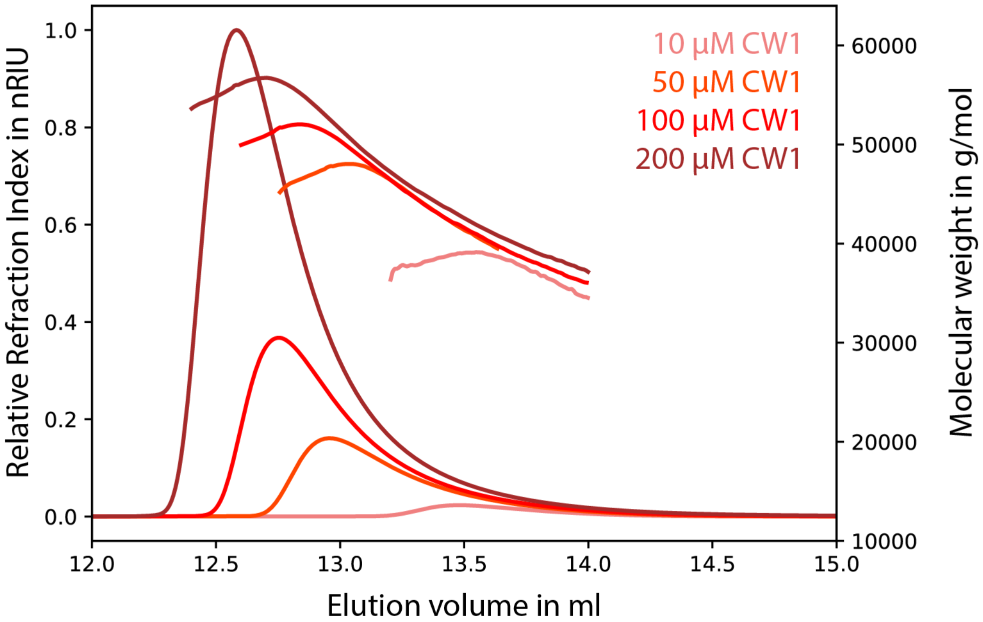

CW1 P-domains were applied to a Superdex In 75 10/300 GL size exclusion column at different (monomeric) protein concentrations (100 µl each). Increasing protein concentrations lead to a shift to lower elution volumes, i.e. higher apparent molecular weights, and increased peak tailing, indicating an concentration-dependent increase in dimeric P-domains accompanied by a noticeable dissociation of dimers during the chromatographic separation. Multi-angle light scattering corroborates this finding: at low protein concentrations P-domains elute mostly as monomers (theoretical molecular weight: 33.2 kDa). At higher protein concentrations, higher molecular weights are found. However, the light scattering curve is not constant but decays towards the molecular weight of P-domain monomers due to a shift in monomer/dimer equilibrium during the run.

Fig. S6: The MNV P-domain has a weak tendency to form dimers.

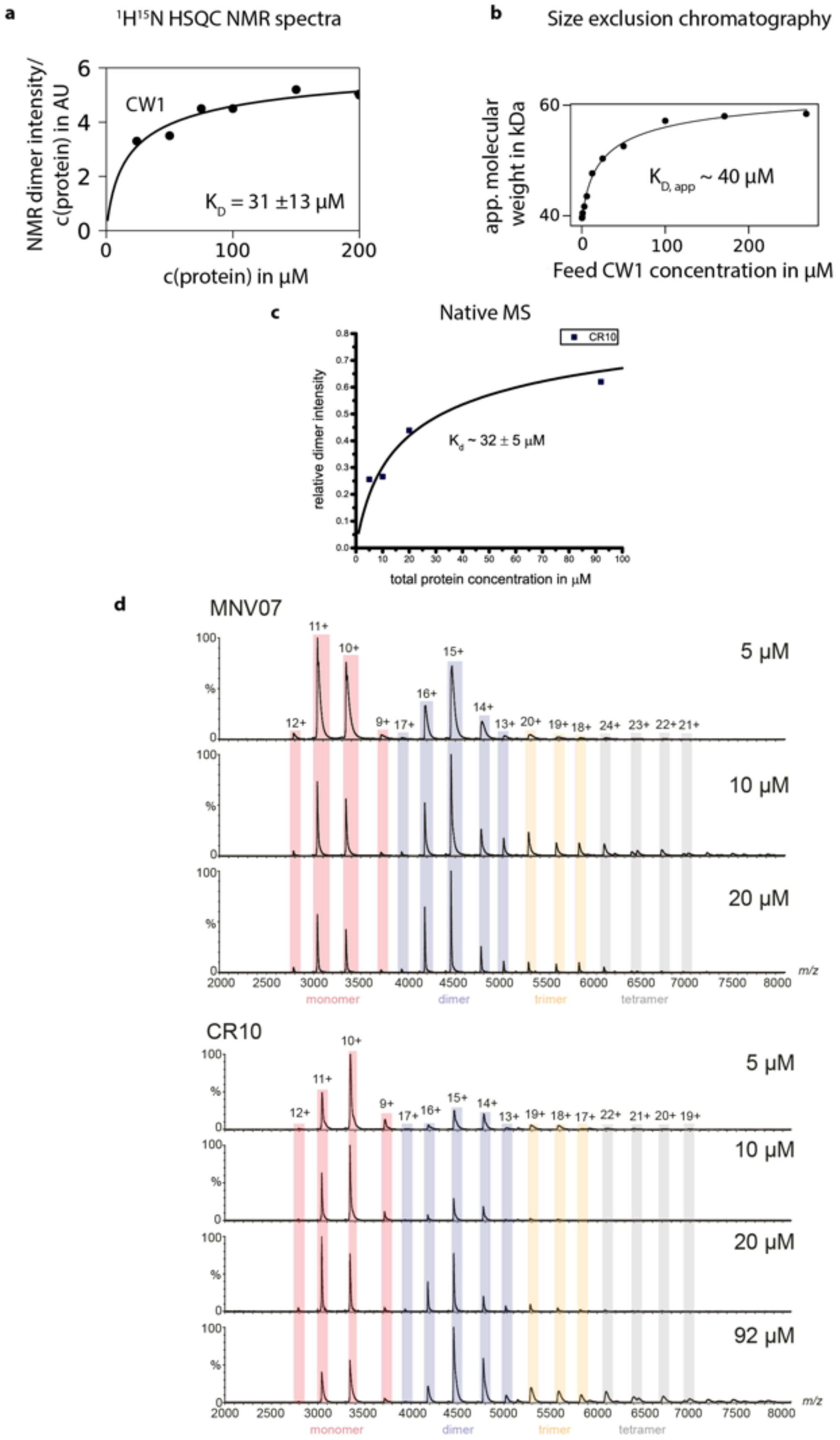

a The dissociation constant of the dimerization reaction was estimated based on the signal intensity of dimer signals (cf. Fig. 1b) in $^{1}H,^{15}N$ TROSY HSQC spectra of [U-$^{2}H,^{15}N$] labeled MNV CW1 P-domains at different total protein concentrations. The signal intensities of 9 dimer signals (6.9|123.8 ppm, 8.5|130 ppm, 10.0|130.2 ppm, 8.8|125.6 ppm, 8.6|126 ppm, 7.5|131 ppm, 8.8|128.9 ppm, 9|112 ppm, 8.1|128.6 ppm) were averaged and fitted against the law of mass action. **b** Fitting the apparent molecular weight from concentration-dependent SEC runs (Fig. 1c) against the law of mass action yields an approximate dissociation constant of 40 µM. Taking into account sample dilution of up to factor of 10 (10 µl injection volume but 100 µl peak base width), the lower K_D limit is 4 µM. **c** Monomer-dimer ratios at different protein concentrations as determined by native MS were fitted to the law of mass action (Eq. S7) to yield the dimerization dissociation constant for the MNV strain CR10. **d** Native mass spectra of MNV07 and CR10 P-domains at different P-domain concentrations.

Fig. S7: Assignment of ^{1}H,^{13}C cross peaks in a methyl-TROSY spectrum of MIL^{ProS}VP^{roS}A-labeled sample of MNV CW1 P-domain saturated with GCDCA.

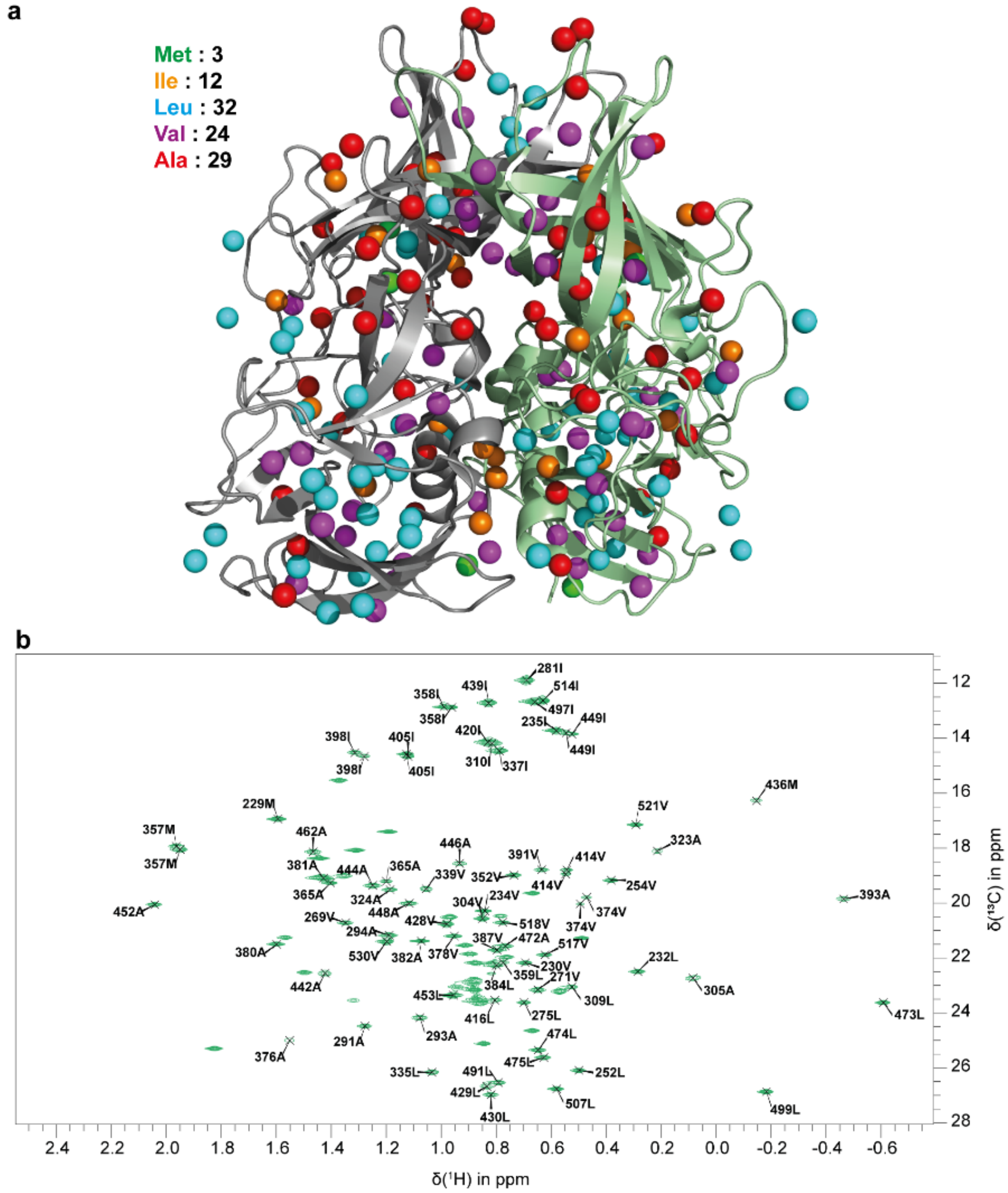

a MNV P-domain crystal structure (PDB 6e47). Methyl groups are highlighted as colored spheres and are distributed over the entire protein. **b** ^{1}H,^{13}C HMQC spectrum of 500 µM MIL^{ProS}V^{ProS}A-labeled MNV P-domain in presence of 700 µM GCDCA measured at 600 MHz (Bruker Avance III HD with TCI cryogenic probe). Assigned resonances are denoted with a cross and their corresponding amino acid type and number. Figure reproduced under creative commons attributions license from [4].

Fig. S8: Methyl TROSY cross peaks reflect slow to fast-to-intermediate exchange upon titration of a sample of MIL^{ProS}V^{ProS}A-labeled P-domain of MNV CW1 with GCDCA.

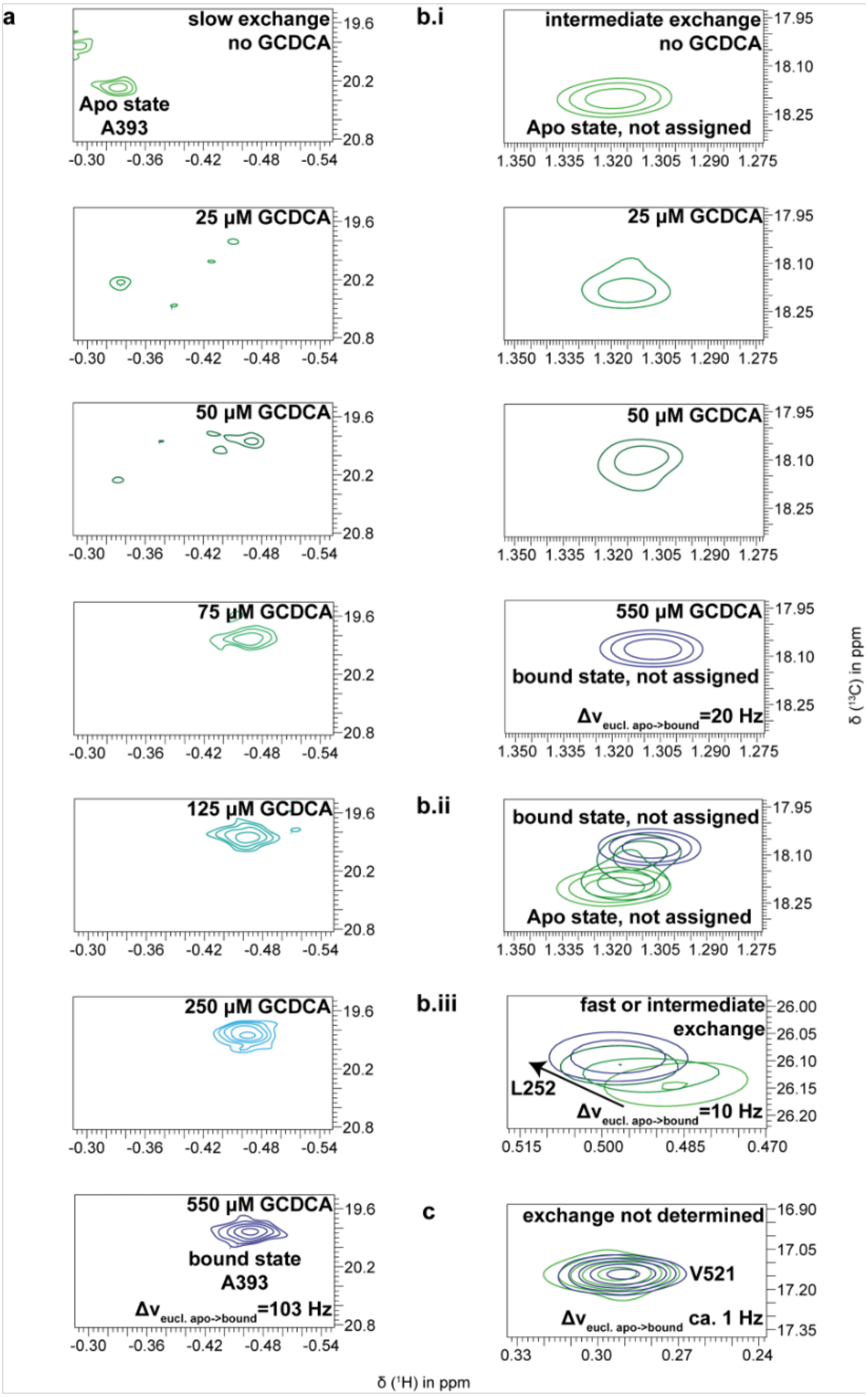

a Slow exchange: The Euclidian distance $\Delta\nu_{Eucl}$ (103 Hz) between free and bound state is much larger than the GCDCA exchange rate, leading to separate signals for the bound and the free state of the P-domain. **b.i-ii** Slow-to-intermediate exchange: The Euclidian distance $\Delta\nu_{Eucl}$ (20 Hz) between free and bound state is slightly larger than the exchange rate, causing signals of low intensity (CSP) being visible during the titration. **b.iii** Fast-to-intermediate exchange: Signals shift during titration (CSPs) with slightly varying intensities, indicating that Euclidian distance $\Delta\nu_{Eucl}$ (< 10 Hz) approach the exchange rate. **c** The Euclidian distance $\Delta\nu_{Eucl}$ is much below 1σ and, therefore, it this peak would be classified as showing no CSP. Qualitatively, it can be said that the exchange rate should be larger than 10 Hz and certainly below 103 Hz, which agrees with the value of 26 Hz as given in Table S2.

Fig. S9: 2D line shape analysis of $^{1}H,^{13}C$ HMQC spectra of MIL^{proS}V^{proS}A-labeled MNV CW1 P-domain at increasing protein concentrations.

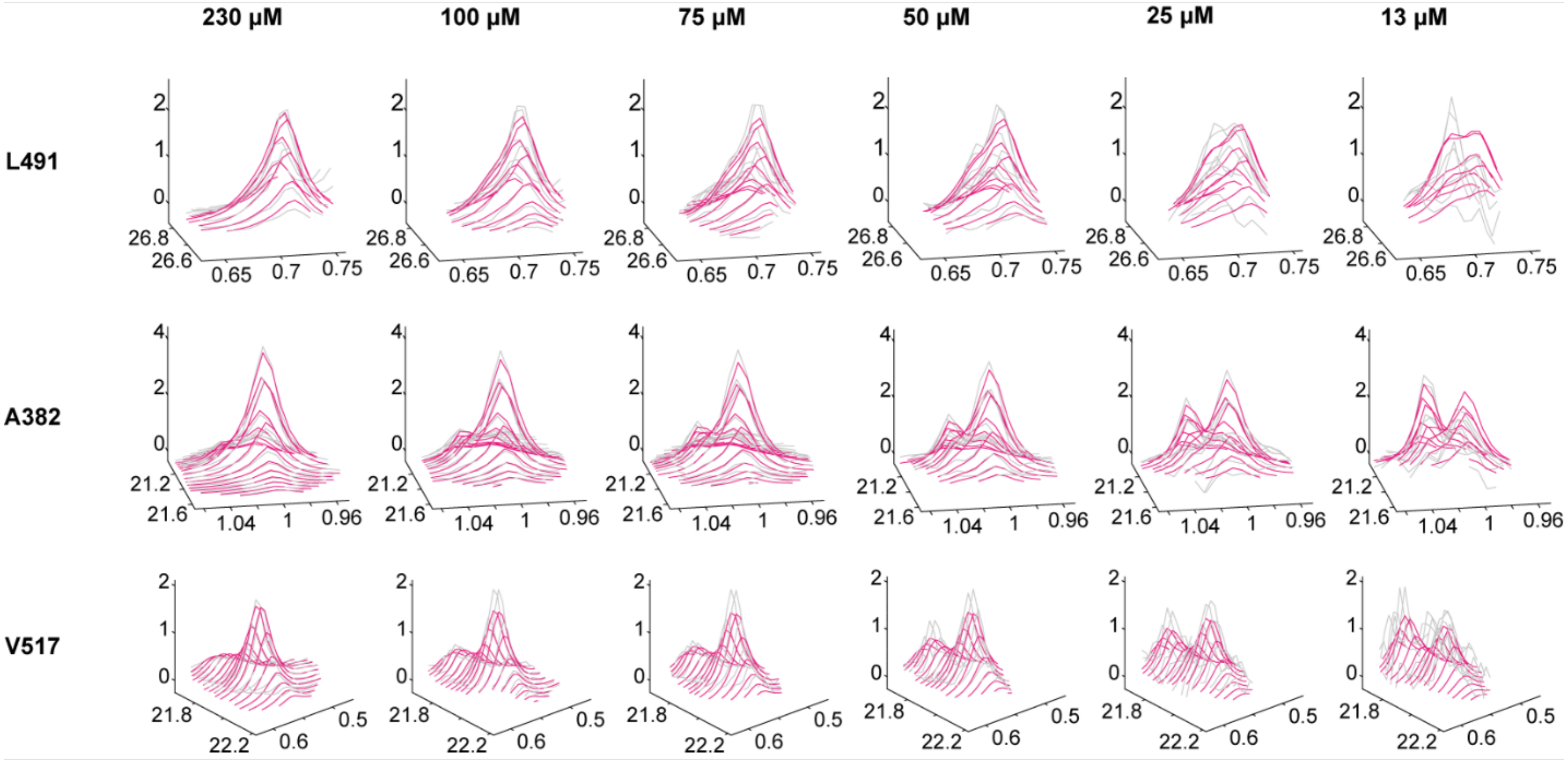

Global line shape analysis of a series of concentration dependent methyl TROSY ($^{1}H,^{13}C$ HMQC) spectra using TITAN[5] yielded a fitted set of cross peaks. The reproducibility of the global fitting improves with the number of peaks used. Therefore, we included not assigned cross peaks in the global fitting when the corresponding peaks were well defined, allowing to fit a set of 13 monomer-dimer signal pairs. It should be noted that in principle this analysis does not depend on any assignments. Experimental and fitted cross peak are shown in grey and magenta, respectively, reflecting the good quality of the fit. TITAN analysis results in a dissociation constant $K_{D,Dimer}$ of 6.9 ± 0.6 µM and a dissociation rate constant $k_{off,Dimer}$ of 1.25 ± 0.21 s^{-1}.

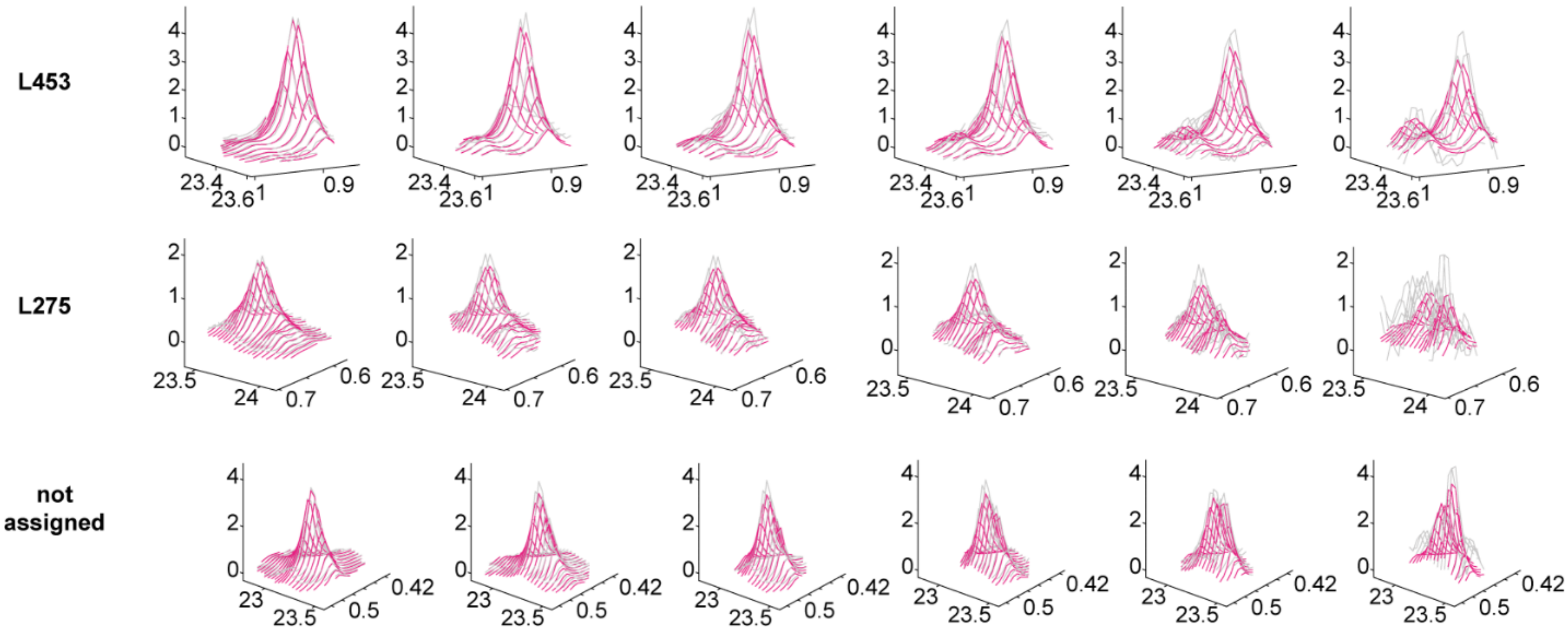

Fig. S9 continued

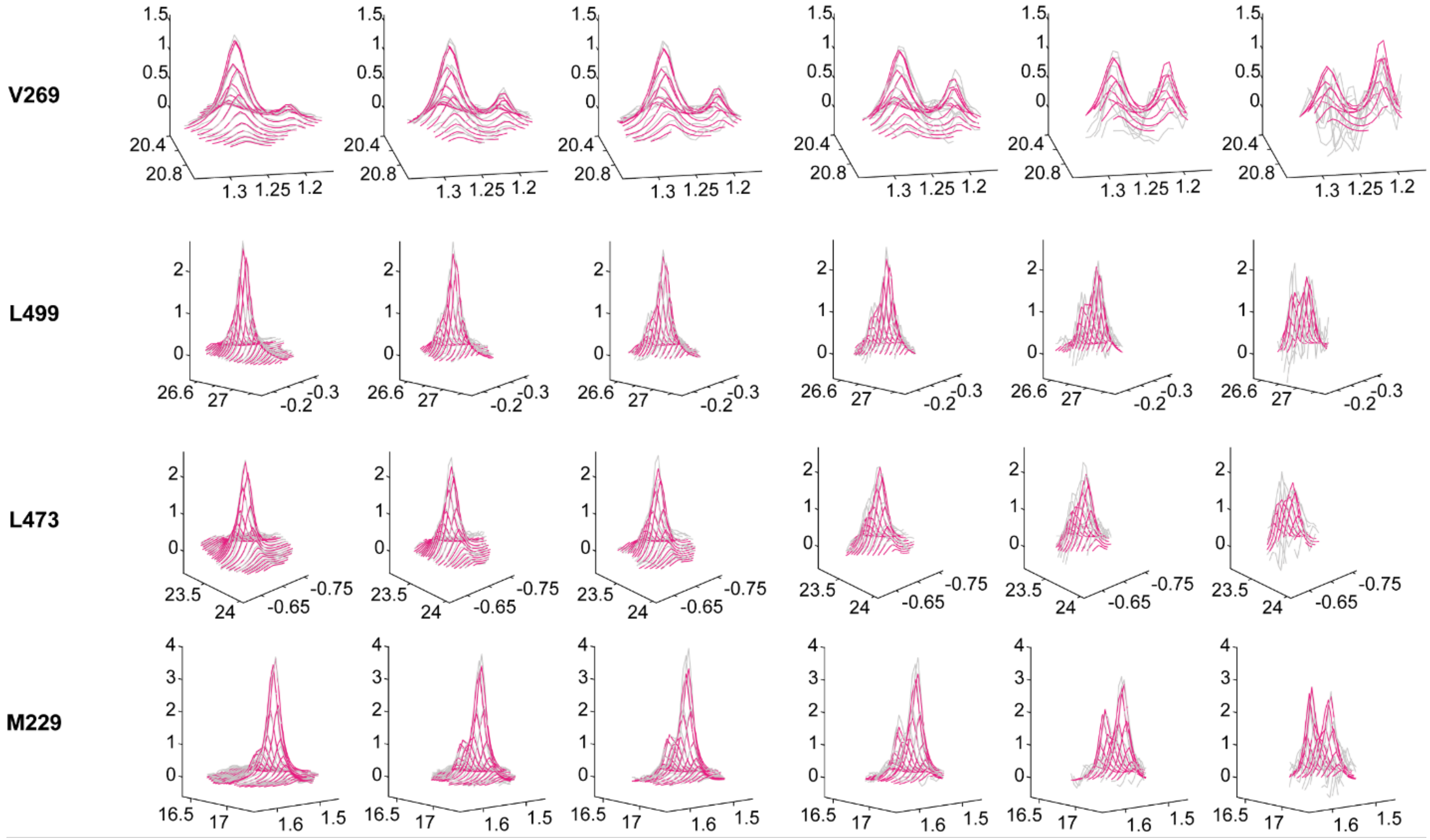

Fig. S9 continued

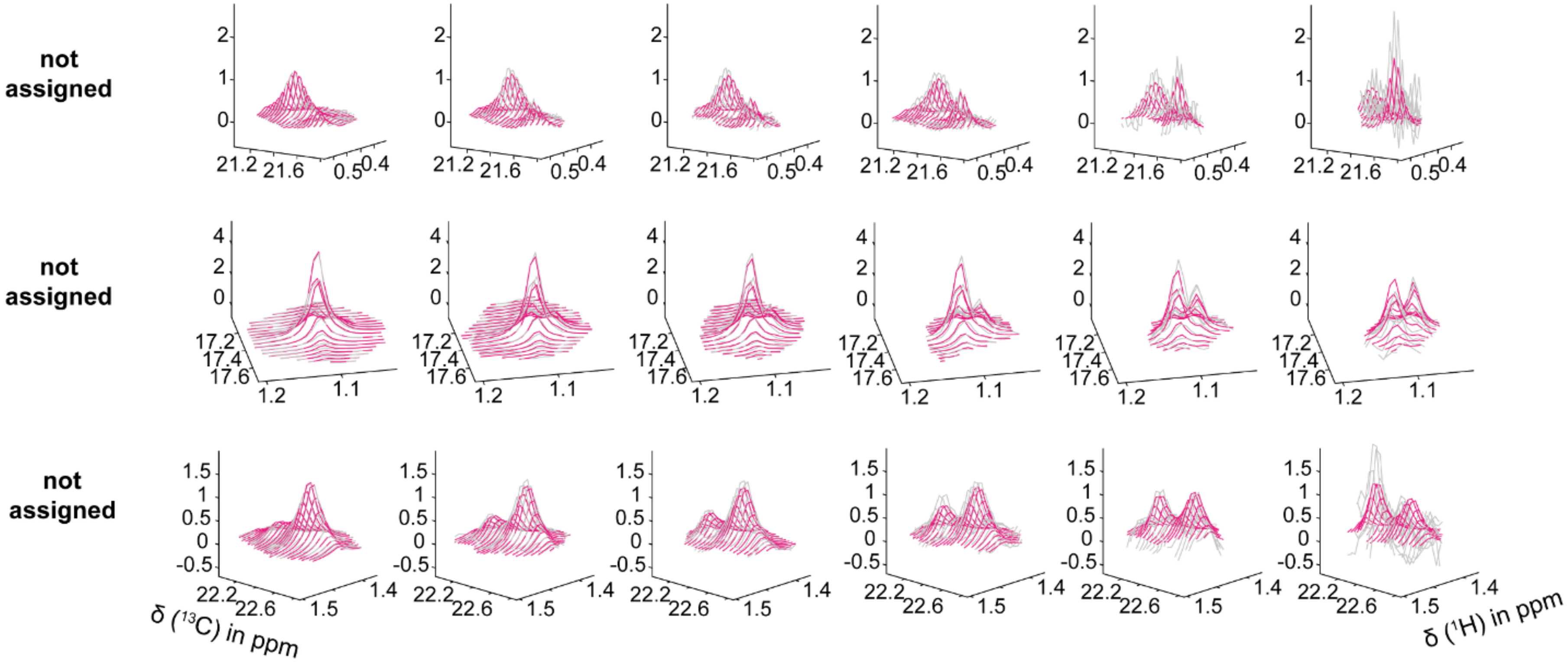

Fig. S9 continued

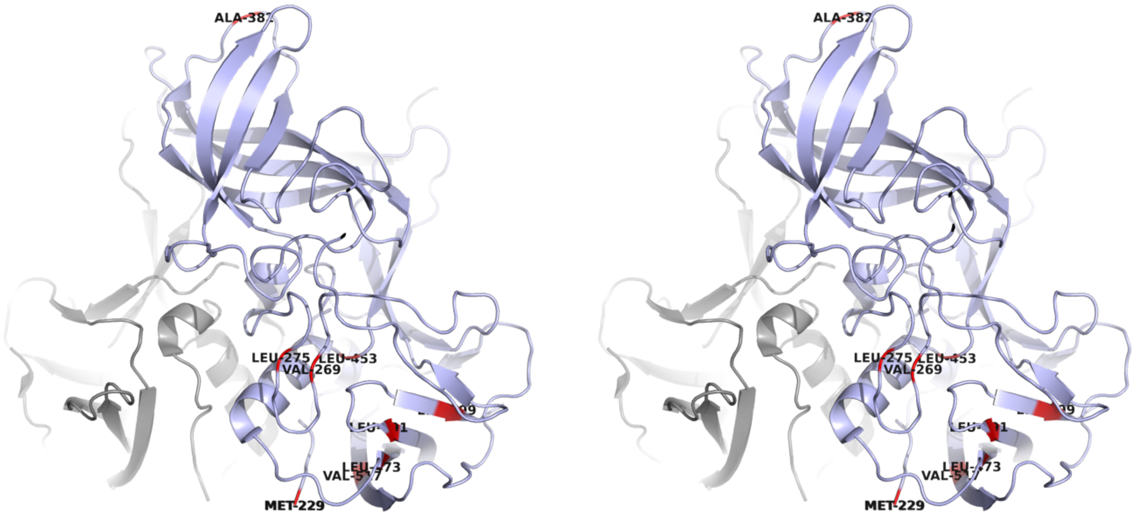

Fig. S9 continued: Crossed-eyed stereo presentation of the MNV P-dimer (PDB 6c6q) with the assigned amino acids used for global line shape analysis highlighted in red. Only shown for one monomer (lightblue), with the other monomer (grey) in the background.

Fig. S10: Comparison of MNV and huNoV P-dimers (GII.4 Saga).

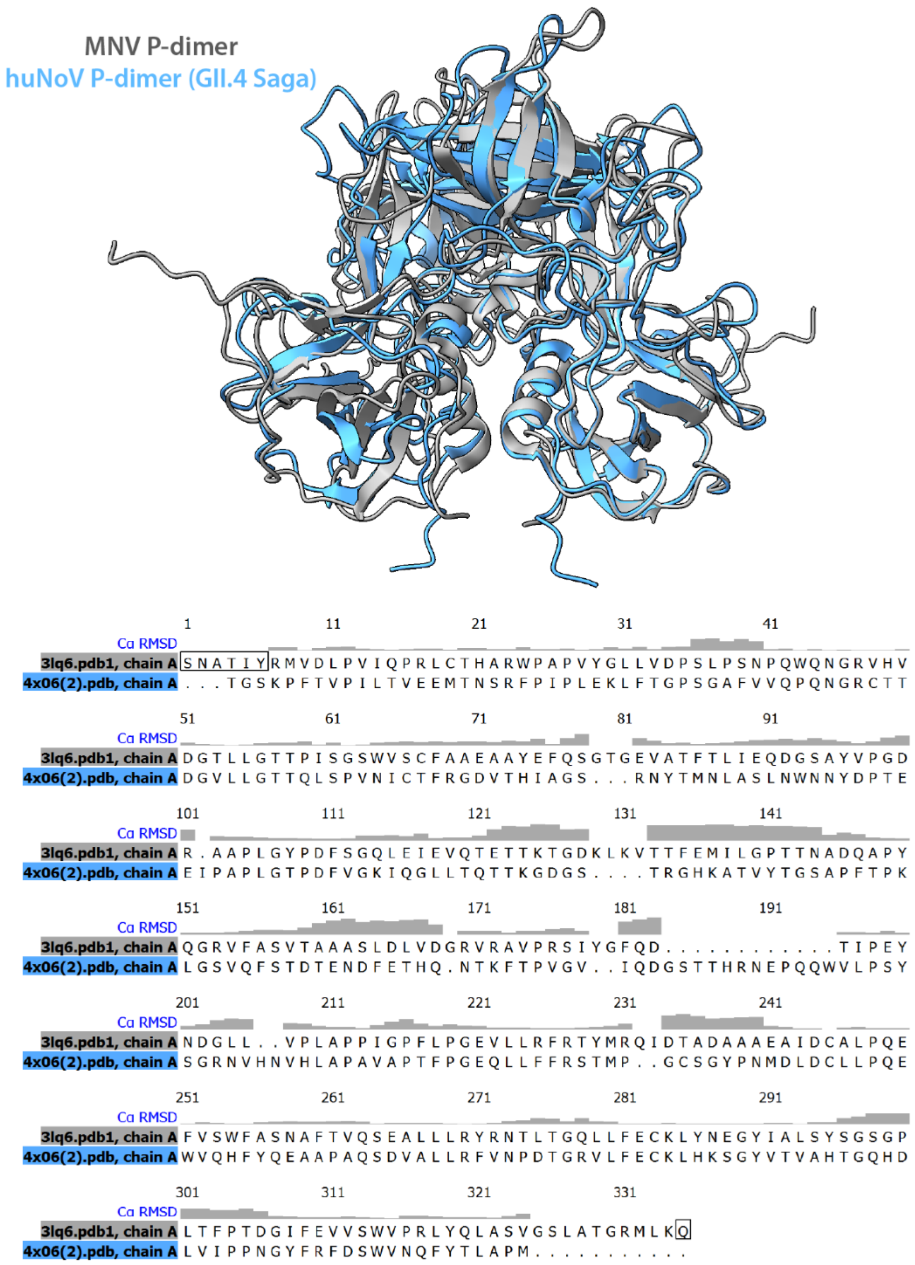

PDB entries 3lq6 (MNV, gray) and 4x06 (huNoV, blue) were structurally aligned using ChimeraX's matchmaker functionality. The overall Cα RMSD is 3.7 Å, while sequence identity after a structure-based multiple sequence alignment is only 31%.

Fig. S11: Increase of thermostability of MNV P-domains and virions in the presence of GCDCA.

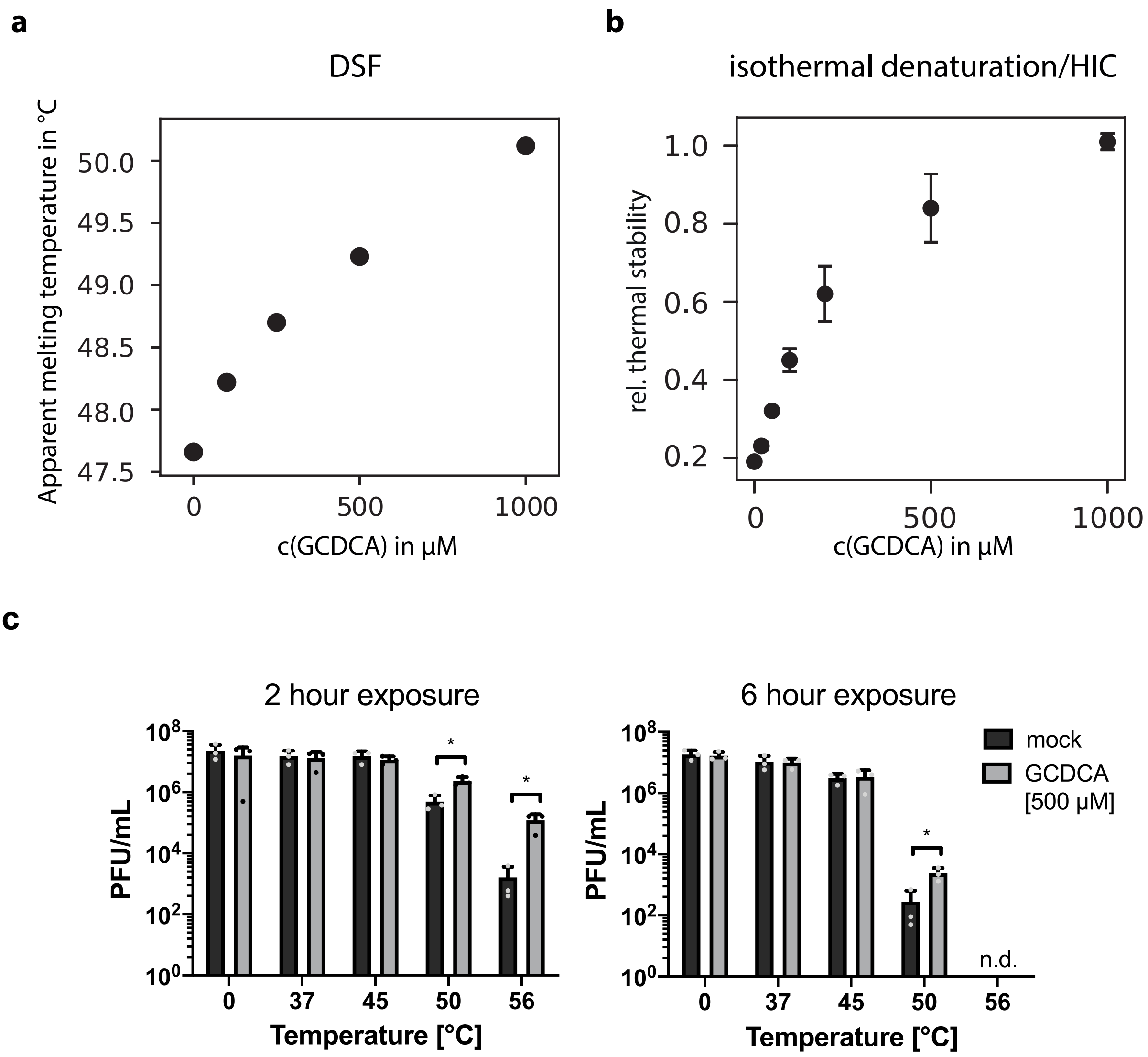

a Apparent protein melting temperatures with varying GCDCA concentrations were obtained by DSF as explained in Fig. 3b. **b** GCDCA-dependent MNV CW1 P-domain stability was confirmed by isothermal protein denaturation for 30 min at 45 °C and analysis of non-denatured P-domain via hydrophobic interaction chromatography (HIC) shows complete protection with 1 mM GCDCA when compared to a non-heat-treated control. HIC experiments were performed as duplicates. The respective percentage of deviation is given as error bars. **c** Increase of thermostability of MNV P-domains in the presence of GCDCA translates to the thermostability of MNV virions. Thermal stability profile of MNV-1.CW3 virions in the presence and absence of the bile acid GCDCA is shown. 1 x 10^7 plaque-forming unit (PFU) of MNV-1.CW3 were incubated in the presence or absence of 500 µM GCDCA for 2 or 6 hours at indicated temperatures between 0 and 56 °C. Duplicate plaque assays were performed for three independent assays. Bars represent the mean (n=3) ± standard deviation (SD). Statistical analysis was performed using the unpaired *t*-test (p-value < 0.05). The * indicates significance (p<0.05) comparing titers obtained in the presence or absence of GCDCA. (n.d. = no detectable titer).

Fig. S12: Increase of MNV P-domain thermal stability by ligand binding is specific for GCDCA.

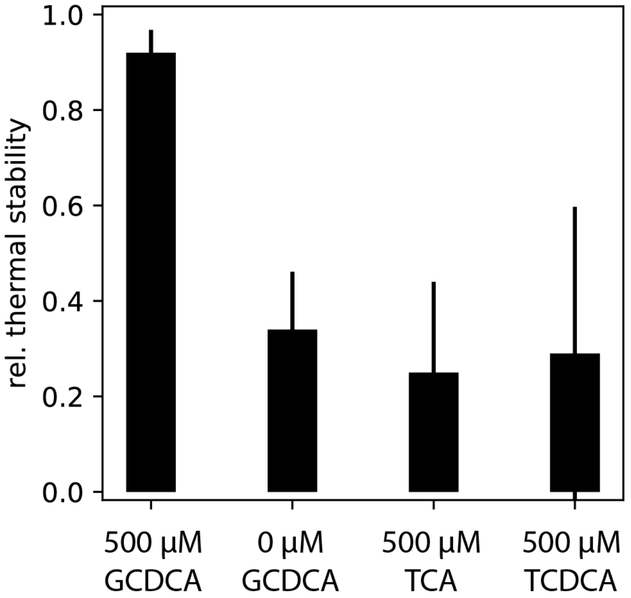

Isothermal denaturation was performed for 30 min at 45 °C and analysis of non-denatured P-domain via HIC was performed by integration of UV absorption at 214 nm. GCDCA = glycochenodeoxycholic acid, TCA = taurocholic acid, TCDCA = taurochenodeoxycholic acid. HIC experiments were performed as duplicates, the respective percentage of deviation is given as error bars.

Fig. S13: Changes in NMR relaxation times indicate altered protein dynamics upon dimerization and ligand binding.

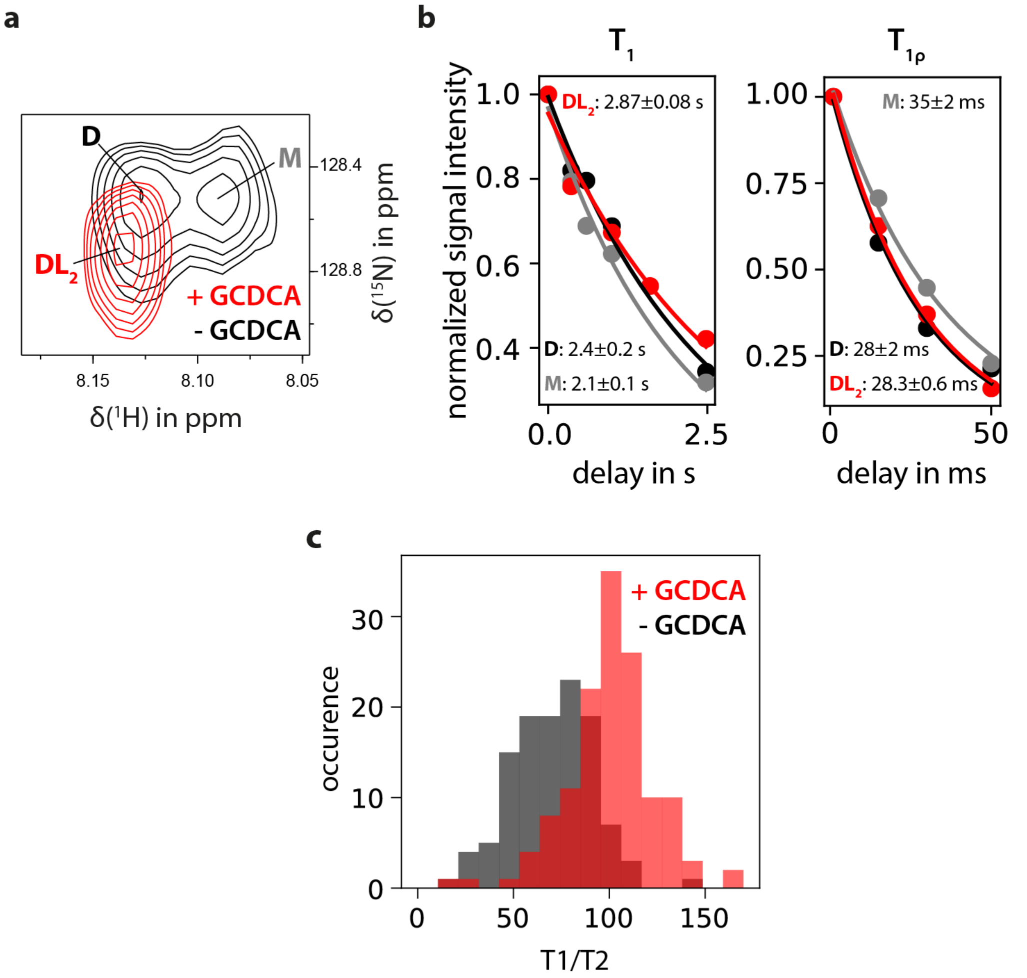

a ^{15}N T_1 and $T_{1\rho}$ relaxation times [6] can be measured for individual amino acid signals in ^{1}H,^{15}N TROSY HSQC spectra of MNV CW1 samples with and without GCDCA. For some spectral regions, signals can be assigned to monomeric (M), dimeric (D), or ligand-bound, dimeric (DL_2) protein species. **b** Relaxation times can be obtained by curve fitting of decaying signal intensities with increasing delays during which relaxation can occur. Relaxation times were found to vary between the different protein species, e.g., between the signals corresponding to the different states of one amino acid shown in (**a**). **c** The ratio T_1/T_2 is sensitive towards protein dynamics on the ps-ns time scale. Global analysis of T_1/T_2 for a high number of amino acids in the absence and in the presence of GCDCA (n=116 and 134, respectively) reveals pronounced differences between the unbound and bound protein states (**c**). ^{15}N T_1 and $T_{1\rho}$ relaxation experiments were measured with a sample containing 230 µM [U-^{2}H,^{15}N]-labeled MNV1 P-domain in the absence of GCDCA and with a sample containing 150 µM protein and 500 µM GCDCA. Samples were prepared in 20 mM sodium acetate, 100 mM NaCl (pH 5.3) and contained 10 % D_2O.

Fig. S14: MNV P-domain proteins titrated with GCDCA revert to their apo state during size exclusion chromatography (SEC).

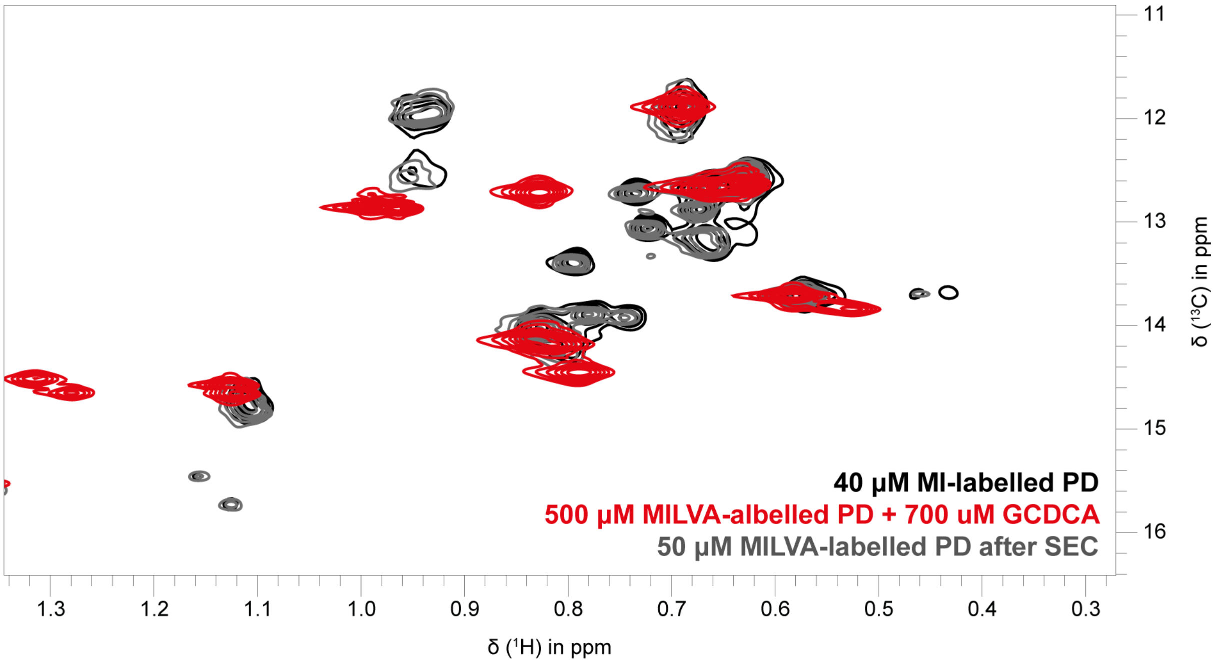

The black spectrum shows the isoleucine region of MI-labelled apo P-domain. The red spectrum shows the isoleucine region of MILVA-labelled P-domain in presence of saturating amounts of GCDCA. The protein was subjected to SEC and another spectrum was acquired (grey). The resonances of the apo P-domain show the same fingerprint as the re-purified protein.

Fig. S15: Overlay of sections of methyl TROSY spectra of MIL^{proS}V^{proS}A-labeled MNV CW1 P-domain in the presence and absence of GCDCA.

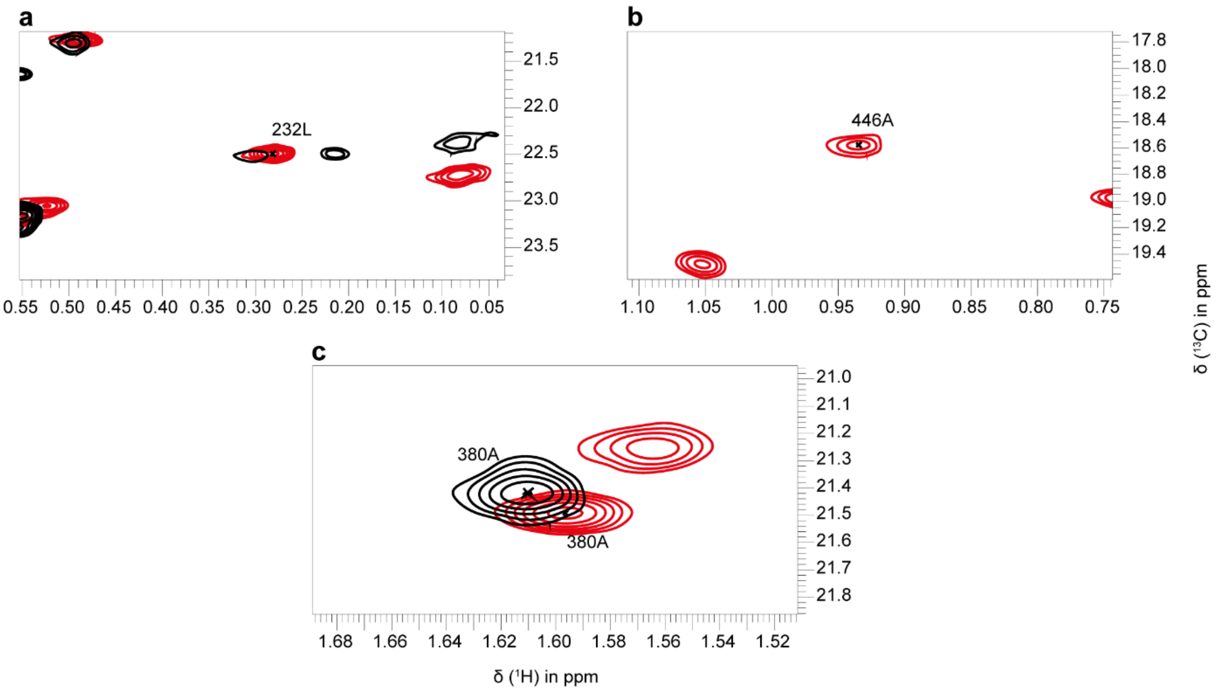

Peaks of methyl TROSY spectra of MIL^{proS}V^{proS}A-labeled MNV CW1 P-domains with GCDCA bound are colored red. The apo form is colored black. **a** The resonance signal assigned to L232 in the bound form cannot be identified unambiguously in the apo-form, where a resonance signal is found in close vicinity but is not well resolved. Therefore, CSPs cannot be determined. **b** The cross peak for A446 is an example of well-isolated resonances where no corresponding apo signal can be identified in close vicinity (<30 Hz). We conclude that the apo signal either shows a CSP with a Euclidian distance $\Delta\nu_{Eucl}$ larger than 30 Hz, or the apo-state signal is broadened to the extent that it is not observable. **c** The cross peak for A380 is an example for signals where both, apo and bound state resonances are well resolved. Corresponding CSPs are readily measured and compiled in Table S3.

Fig. S16: Determination of the minimum threshold for significant chemical shift perturbations

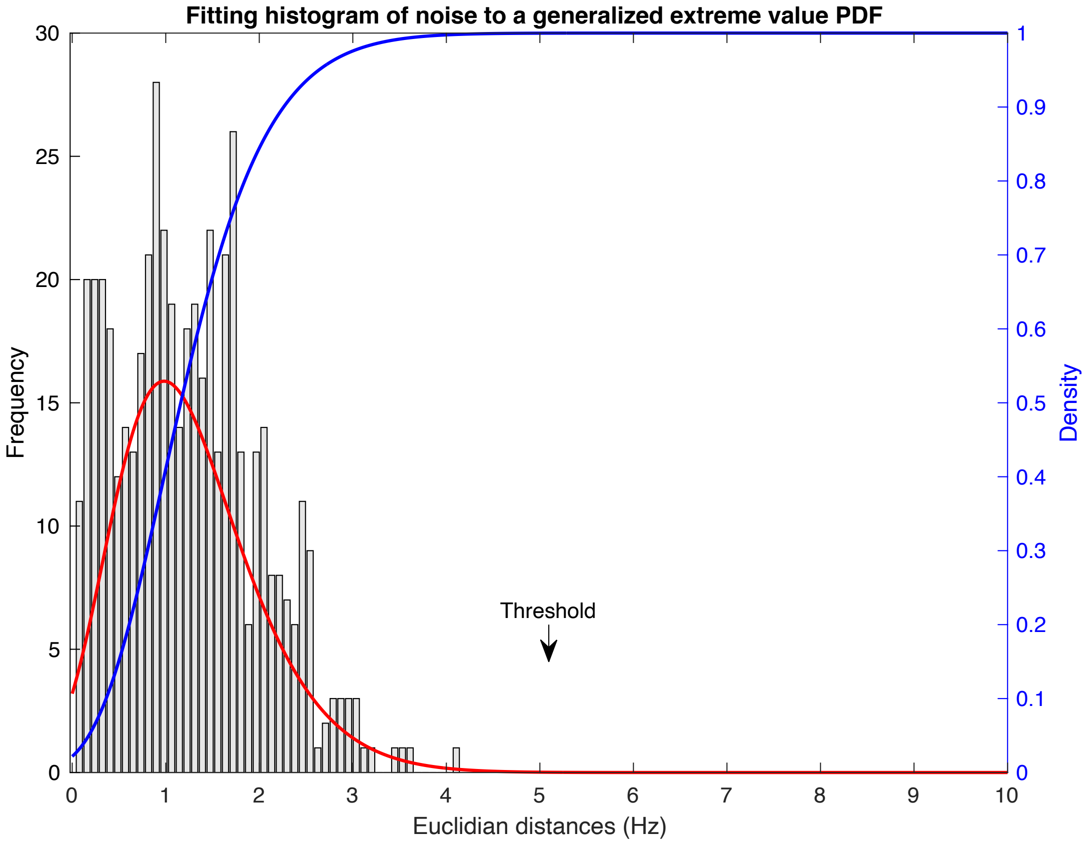

To quantify the precision of chemical shift perturbation measurements we used five samples of MNV CW1 P-domain in the presence of saturating amounts of GCDCA and acquired methyl TROSY spectra. The samples differed in age, concentration, and batch origin. 50 cross-peaks were picked, and Euclidean chemical shift differences were calculated for all possible pairwise permutations of the five measurements affording a 50 x 10 noise matrix. The calculation of the minimum threshold employed fitting a generalized extreme value (GEV) probability density function (red) to the experimental data represented as bars with each bar representing the number (frequency) of Euclidian distances at this point. The blue curve shows the cumulative density function, and the arrow indicates the 99.99% confidence level at a Euclidian distance of 5.1 Hz. The equations and the fitting procedure have been described in detail in ref. [7].

Fig. S17: Histogram showing CSPs for the MNV CW1 P-domain upon addition of GCDCA.

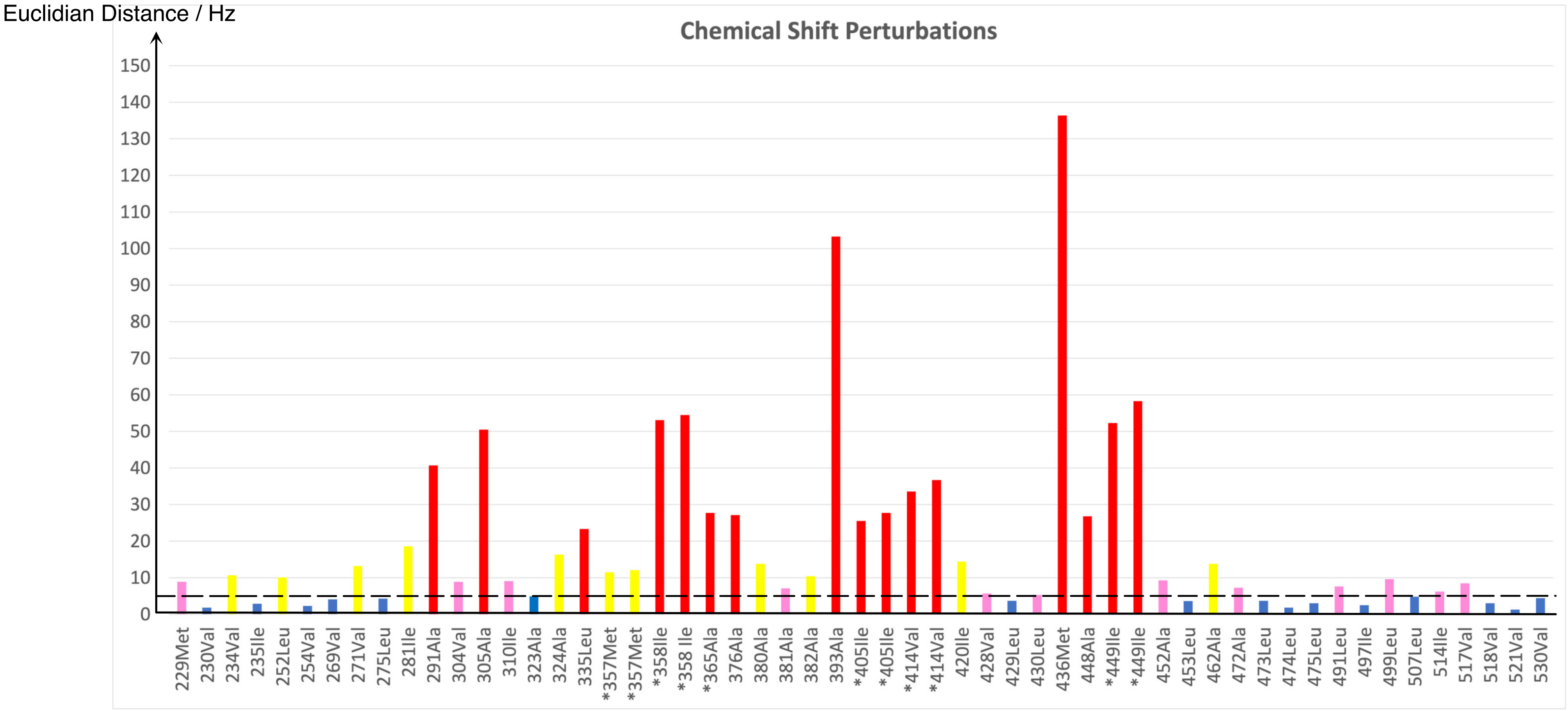

The CSP values have been taken from Table S3. Red bars: Δν > 20 Hz ("large"); yellow bars: 10 Hz < Δν < 20 Hz ("medium"); pale magenta bars: 5.1 Hz < Δν < 10 Hz ("small"). "Small" CSPs are not discussed in the main text but they are above the minimum threshold of 5.1 Hz and thus count as significant (cf. Fig. S16). Blue bars represent non-significant CSPs. The dashed line is marking the threshold (5.1 Hz) for significant CSPs.

Fig. S18: Comparison of GCDCA-induced changes to the MNV P-domain based on crystal structure analysis with NMR-derived chemical shift mapping.

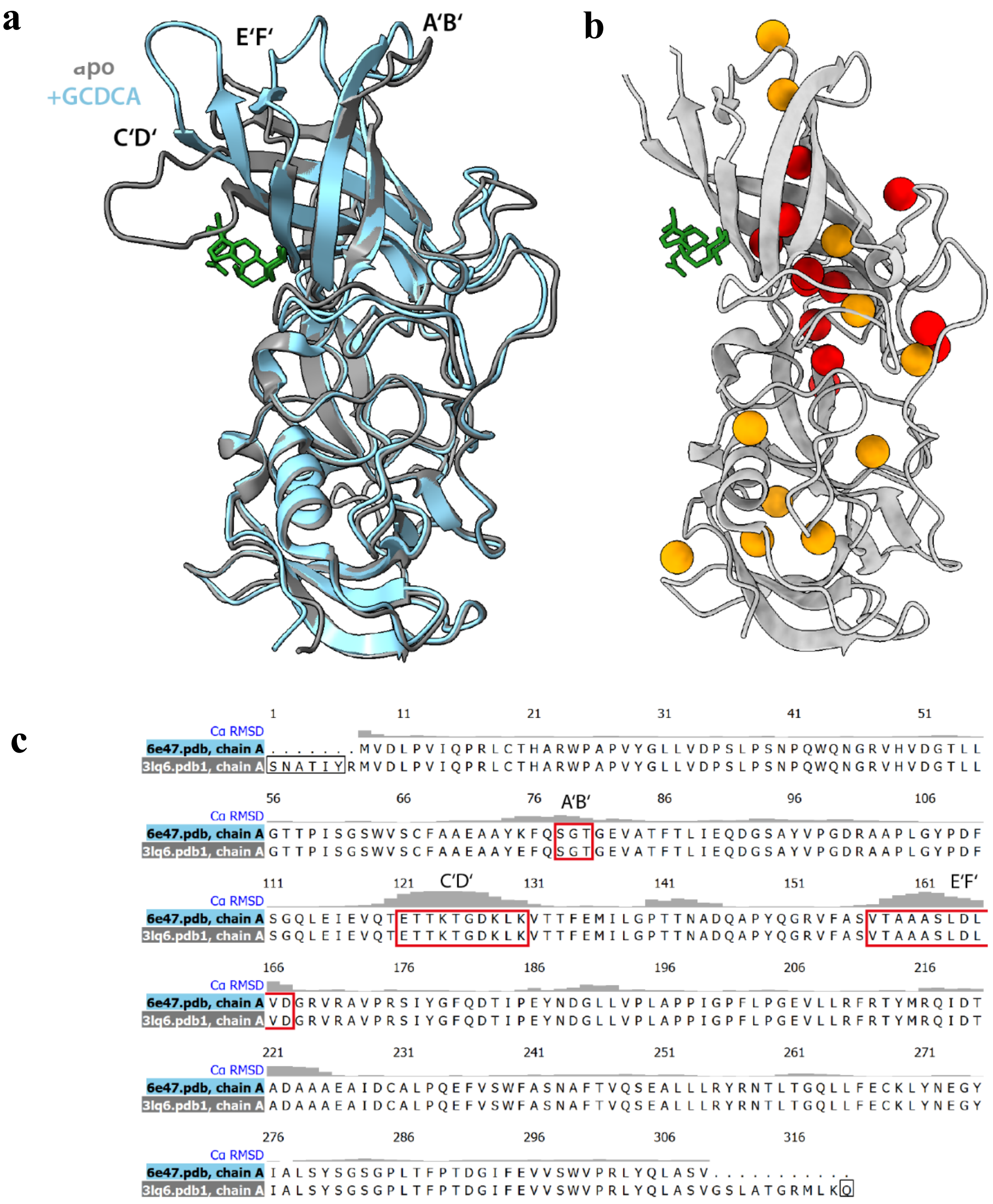

a Overlay of the crystal structure of the apo (gray, PDB 3lq6) and of the ligand-bound (blue, PDB 6e47) form of the MNV P-domain, highlighting associated conformational changes. **b** Chemical shift perturbations maped on the GCDCA-bound form. **c** Crystal structures of the apo (gray, PDB 3lq6) and the ligand-bound MNV P-domain (blue, PDB 6e47) were superimposed using ChimeraX's matchmaker functionality. Per-residue Cα RMSD values are represented as small grey bars above the sequence and are mostly limited to the three indicated surface loops (red boxes). NMR CSP data is reproduced from Fig. 4, showing medium (orange) and large (red) chemical shift perturbations.

Fig. S19: GCDCA but not TCA blocks neutralization of different monoclonal antibodies.

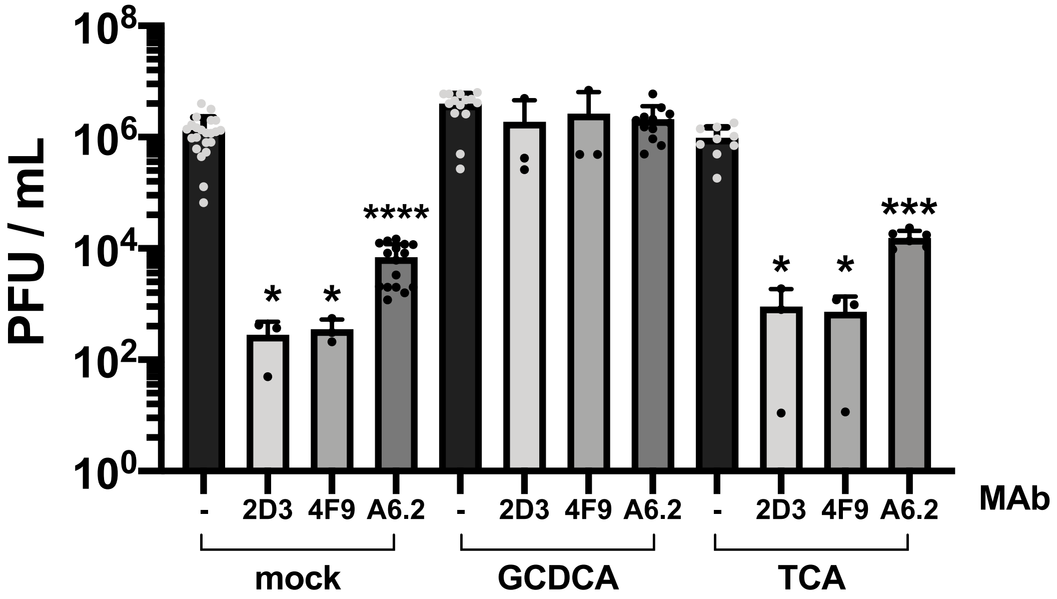

MNV-1.CW3 immune escape from monoclonal antibodies (MAb) 2D3, 4F9, and A6.2 was determined by plaque neutralization assay in the presence or absence of the 500 µM bile acid (GCDCA or TCA) and 2 µg/ml of the respective neutralizing antibody. Plaque assays were performed in duplicate for at least three independent assays and plaque forming units (PFU) per ml were determined. Bars represent the mean (n=3) ± standard deviation (SD). Statistical analysis was performed using the unpaired *t*-test. The * indicates significance ($p<0.05$), *** indicates significance ($p<0.001$), and **** indicates significance ($p<0.0001$) compared to the respective treatment without MAb.

Fig. S20: GCDCA improves infectivity of MNV-1 and prevents neutralization with antibody A6.2

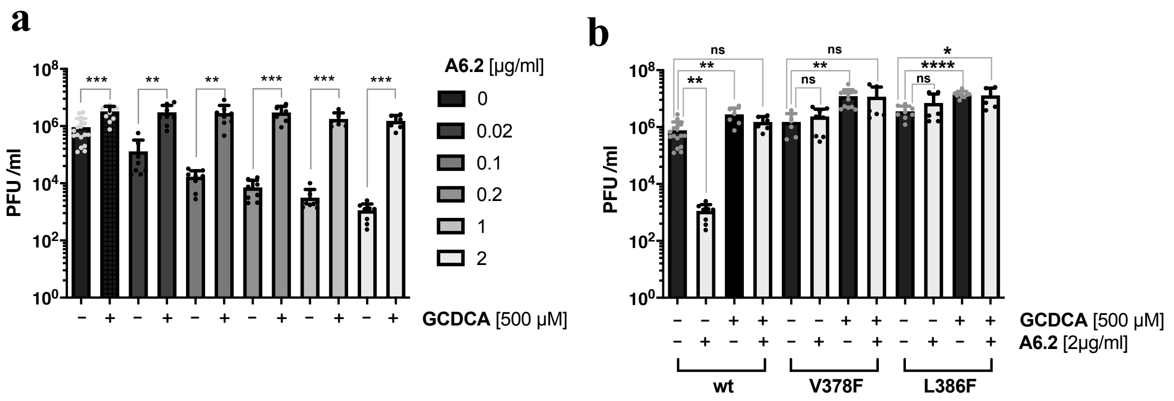

MNV immune escape from monoclonal antibody (MAb) A6.2 was determined by plaque neutralization assay. All plaque assays were performed in duplicate for at least three independent assays and plaque forming units (PFU) per ml were determined. Statistical analysis was performed using the unpaired *t*-test. Bars represent the mean (n=3) ± standard deviation (SD). Statistical analysis was performed using the unpaired *t*-test. The significance is indicated as *(p<0.05), ** (p<0.01), *** (p<0.001), and **** (p<0.0001). **a** Neutralization of $1x10^6$ PFU/ml of MNV-1.CW3 was determined in the presence of increasing monoclonal antibody A6.2 concentrations (0 – 2 µg/ml) and in the presence or absence of 500 µM GCDCA. All concentrations of A6.2 showed significant neutralization (p<0.05; omitted from graph for clarity) when compared to untreated. The significance in the titer difference between GCDCA treated versus untreated samples are shown. **b** Immune escape from monoclonal antibody A6.2 was determined in the absence or presence of bile acid GCDCA (500 µM) for recombinant parental wild-type (MNV-1.CW1), and isogenic descendants MNV-1.CW1(VP1:V378F), and MNV-1.CW1(L386F) containing only respective A6.2 escape mutations[8]. Significant increase in infectivity was observed in the GCDCA treated samples, partially retained even in the presence of neutralizing MAb A6.2.

Fig. S21: Representative sections from $^1H,^{15}N$ TROSY HSQC spectra of MNV CW1 P-domain

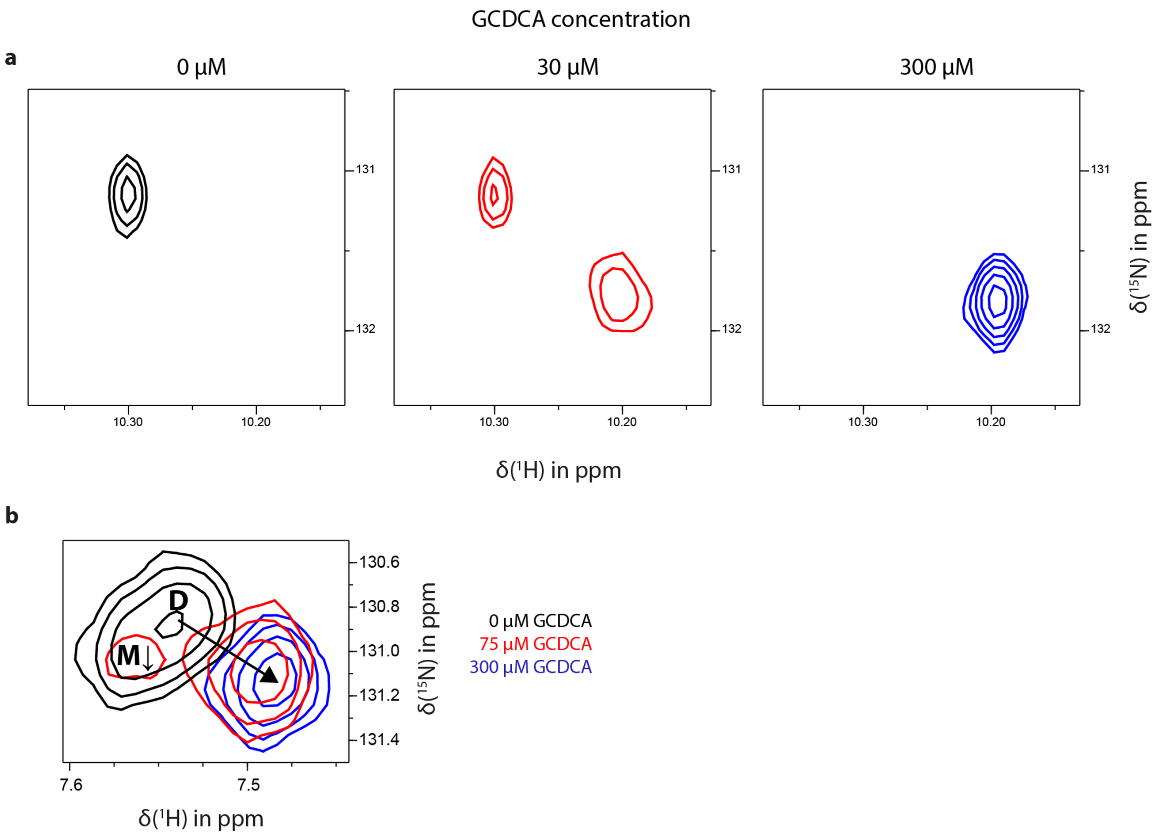

Representative sections from $^1H,^{15}N$ TROSY HSQC spectra of 100 µM [*U*-$^2H,^{15}N$]-labeled MNV CW1 P-domain. With increasing GCDCA concentrations both, signals in slow exchange (**a**, $\Delta\nu = 60$ Hz), and signals in intermediate exchange (**b**, $\Delta\nu = 30$ Hz) are observed. Signals attributable to the monomer (M) disappear with increasing GCDCA concentrations, whereas some dimer signals (D) display chemical shift perturbations (CSP). Samples were prepared in 20 mM sodium acetate buffer, 100 mM NaCl (pH 5.3).

Fig. S22: Full data set used for the TITAN analysis of GCDCA binding

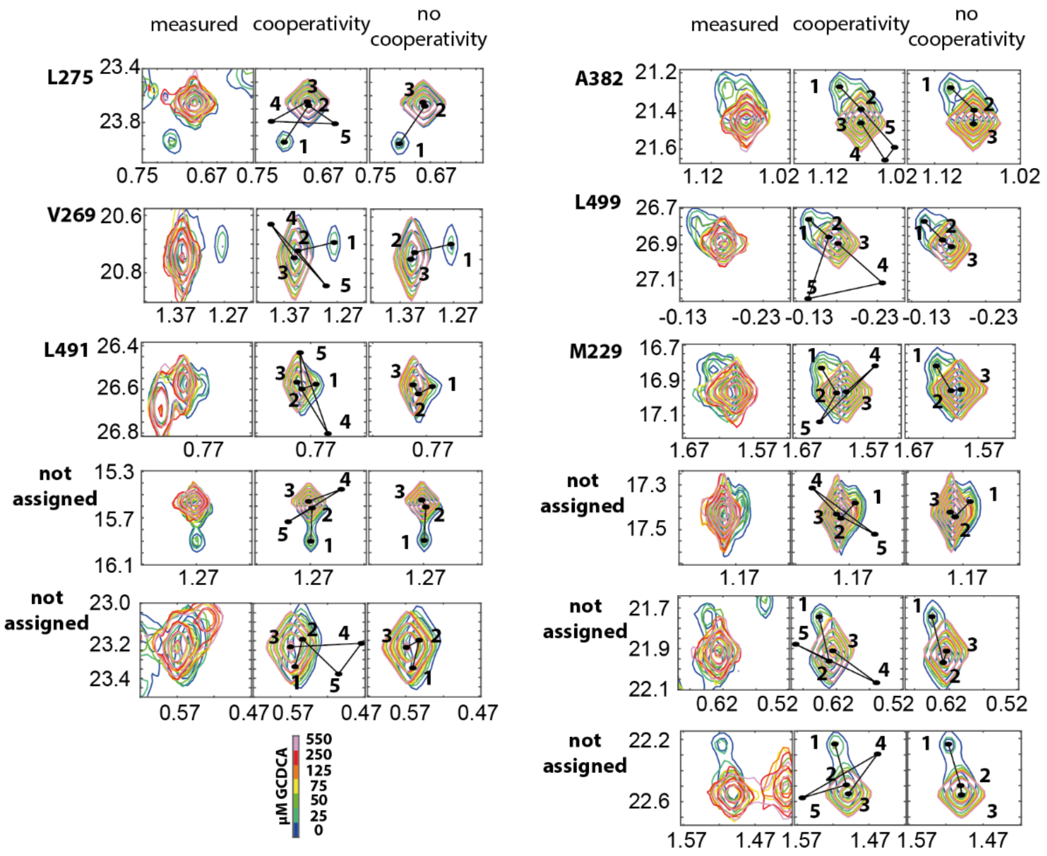

Complete set of cross peaks used for the TITAN analysis of GCDCA binding to the MNV CW1 P-domain. The first column shows the sections from the methyl TROSY experiment as measured. The second column shows the simulated cross peaks using a cooperative binding model for fitting, and the third column contains the simulated cross peaks resulting from a binding model without cooperativity (see Fig. 5 for more details).

Supplementary Tables

Tab. S1: Amino acid sequences of norovirus P-domain proteins studied.

Strain	GenBank ID	Additional N-terminal aa
CW1	aa 228-530 of entry DQ285629	GP
MNV07	aa 228-530 of entry AET79296	GPGS
CR10	aa 228-530 of entry ABU55613	GPGS
Saga2006	aa 225-530 of entry AB447457	GPGS

Additional N-terminal amino acids have been added to create the enzymatic cleavage site.

Table S2: Dissociation constants and rate constants for dimerization of P-domains and binding of GCDCA to P-dimers.

Norovirus strain	P-domain dimerization			GCDCA binding			Experiment
	k_{on} / M^{-1} s^{-1}	k_{off} / s^{-1}	$K_{D,Dimer}$ / µM	k_{on} / M^{-1} s^{-1}	k_{off} / s^{-1}	$K_{D,GCDCA}$ / µM	
**MNV.CW1**	1.81×10^5	1.25 ± 0.21	6.9 ± 0.6	2.46×10^6	25.80 ± 3.50	10.5 ± 0.5	Methyl TROSY / TITAN analysis (Fig. S9)
			31 ± 13				^{1}H,^{15}N HSQC TROSY, CSPs (Fig. S6a)
			4 - 40				Concentration-dependent SEC runs (Fig. 1c and Fig. S6b)
**MNV.CR10**			32 ± 5				Native MS (Fig. S6c)
**MNV.CW3**						6	ITC (see ref. [9])
**GII.4 Saga**		1.5×10^{-6}					IEX (cf. Fig. 5)
						1500	^{1}H,^{15}N HSQC TROSY, CSPs (see ref. [10])

Tab. S3: Transfer of assignments of ^{13}C-methyl groups for the GCDCA-bound form of MNV CW1 P-dimers to the apo-form.

The assignments of GCDCA-bound P-dimers[4] were transferred to the apo P-domain using a nearest neighbor approach as explained in the manuscript. Chemical shifts of resonances where a transfer of assignment was possible are tabulated along with the resulting CSPs. CSPs were calculated as Euclidian distances $\Delta\nu_{Eucl}$ and as distances $\Delta\nu_H$ and $\Delta\nu_C$ in the ^{1}H and ^{13}C dimensions, respectively. Light grey rows signify resonance signals where a transfer of assignment was not possible due to signal crowding and/or line broadening. Dark grey rows highlight peaks that are well isolated (no spectral crowding) in the GCDCA-bound form. However, no corresponding signal of the apo-form can be identified, suggesting that the apo-signal is either outside a Euclidian distance range of ca. 30 Hz, or it is broadened due to unfavorable exchange dynamics between monomers and dimers in the apo-form. The classification of resonance signals is illustrated in Figs. S16 and S17.

	Chemical shifts δ /ppm				CSPs / Hz		
	Apo		GCDCA bound				
Amino acid	^{1}H	^{13}C	^{1}H	^{13}C	$\Delta\nu_{Eucl}$	$\Delta\nu_H$	$\Delta\nu_C$
229Met	1.61	16.9	1.59	16.9	8.9	8.8	1.3
230Val	0.69	22.2	0.69	22.2	1.8	0.2	1.8
232Leu	n.d.	n.d.	0.28	22.5	n.d.	n.d.	n.d.
234Val	0.84	20.2	0.84	20.3	10.7	1.7	10.5
235Ile	0.58	13.7	0.58	13.7	2.9	2.9	0.1
252Leu	0.49	26.1	0.5	26.1	10.1	6.3	7.9
254Val	0.38	19.2	0.38	19.2	2.3	0.3	2.3
269Val	1.34	20.7	1.35	20.7	4.1	2.8	3
271Val	0.63	23.1	0.65	23.2	13.2	11.2	6.9
275Leu	0.7	23.6	0.7	23.6	4.3	0.7	4.3
281Ile	0.69	12	0.69	11.9	18.6	4.4	18
291Ala	1.21	24.6	1.28	24.5	40.7	36.9	17.3
293Ala	n.d.	n.d.	1.08	24.2	#	n.d.	n.d.
294Ala	n.d.	n.d	1.19	21.2	n.d.	n.d.	n.d.
304Val	0.85	20.6	0.85	20.6	8.9	1.4	8.8
305Ala	0.08	22.4	0.08	22.7	50.5	1.2	50.4
309Leu	n.d.	n.d.	0.52	23.1	n.d.	n.d.	n.d.
310Ile	0.81	14.2	0.81	14.2	9.1	2.4	8.8
323Ala	0.22	18	0.21	18.1	4.9	3.9	3
324Ala	1.16	19.5	1.18	19.5	16.3	15	6.3
335Leu	0.99	26.2	1.03	26.1	23.3	21.8	8.2
337Ile	n.d.	n.d.	0.79	14.4	#	n.d.	n.d.
339Val	n.d.	n.d.	1.05	19.5	#	n.d.	n.d.
352Val	n.d.	n.d.	0.73	19	#	n.d.	n.d.
*357Met	1.94	18	1.95	18	11.5	7.3	8.9
*357Met	1.95	17.9	1.96	17.9	12.1	5	11
*358Ile	0.95	12.5	0.96	12.9	53.1	3.6	53
*358 Ile	0.95	12.5	0.99	12.9	54.5	20.7	50.5
359Leu	n.d.	n.d.	0.77	22.2	n.d.	n.d.	n.d.
*365Ala	n.d.	n.d.	1.4	19.3	n.d.	n.d.	n.d.

*365Ala	1.22	19.1	1.2	19.2	27.7	14.8	23.4
*374Val	n.d.	n.d.	0.47	19.8	n.d.	n.d.	n.d.
*374Val	n.d.	n.d.	0.49	20	n.d.	n.d.	n.d.
376Ala	1.54	24.8	1.55	25	27.1	3.3	26.9
378Val	n.d.	n.d.	0.95	21.2	#	n.d.	n.d.
380Ala	1.61	21.4	1.6	21.5	13.8	8.3	11
381Ala	1.43	19.1	1.43	19.1	7.1	0.3	7.1
382Ala	1.07	21.3	1.07	21.4	10.4	0.4	10.3
384Leu	n.d.	n.d.	0.8	22.3	n.d.	n.d.	n.d.
387Val	n.d.	n.d.	0.8	21.7	n.d.	n.d.	n.d.
391Val	n.d.	n.d.	0.63	18.8	#	n.d.	n.d.
393Ala	-0.33	20.3	-0.47	19.8	103.3	80.3	65
*398Ile	n.d.	n.d.	1.31	14.5	#	n.d.	n.d.
*398Ile	n.d.	n.d.	1.27	14.7	#	n.d.	n.d.
*405Ile	1.1	14.8	1.12	14.7	25.5	8.2	24.1
*405Ile	1.11	14.8	1.12	14.6	27.7	8.2	26.4
*414Val	0.53	19	0.54	18.8	33.6	6.1	33.1
*414Val	0.54	19.2	0.54	18.9	36.7	0.4	36.7
416Leu	n.d.	n.d.	0.8	23.5	n.d.	n.d.	n.d.
420Ile	0.83	14	0.83	14.1	14.4	0.8	14.4
428Val	0.98	20.8	0.98	20.8	5.7	1.6	5.5
429Leu	0.83	26.6	0.83	26.7	3.7	0.8	3.6
430Leu	0.82	27	0.82	27	5.3	4.5	2.8
436Met	0.01	16.9	-0.14	16.3	136.4	91.2	101.4
439Ile	n.d.	n.d.	0.82	12.7	#	n.d.	n.d.
442Ala	n.d.	n.d.	1.42	22.5	#	n.d.	n.d.
444Ala	n.d.	n.d.	1.25	19.4	#	n.d.	n.d.
446Ala	n.d.	n.d.	0.93	18.6	#	n.d.	n.d.
448Ala	1.15	19.9	1.11	20	26.8	19.9	17.9
*449Ile	0.46	13.7	0.54	13.8	52.3	49.5	16.8
*449Ile	0.43	13.7	0.52	13.8	58.3	54.2	21.4
452Ala	2.04	20	2.04	20.1	9.3	1.6	9.1
453Leu	0.96	23.4	0.95	23.4	3.6	1.3	3.4
462Ala	1.46	18.2	1.46	18.1	13.8	4.4	13
472Ala	0.76	21.6	0.77	21.6	7.3	4.7	5.6
473Leu	-0.6	23.6	-0.61	23.6	3.7	3.2	1.9
474Leu	0.65	25.4	0.65	25.4	1.8	1.8	0
475Leu	0.63	25.6	0.63	25.6	3	2.9	0.9
491Leu	0.78	26.6	0.79	26.5	7.6	7.1	2.5
497Ile	0.66	12.7	0.66	12.7	2.5	0.9	2.3
499Leu	-0.17	26.8	-0.18	26.9	9.6	7.5	6.1
507Leu	0.59	26.8	0.58	26.8	4.8	3.9	2.8
514Ile	0.63	12.6	0.63	12.6	6.2	1.7	5.9
517Val	0.63	21.9	0.62	21.9	8.5	3.5	7.8
518Val	0.78	20.7	0.78	20.7	3	1.3	2.8
521Val	0.29	17.1	0.29	17.1	1.3	1.3	0.1
530Val	1.2	21.4	1.2	21.4	4.4	2	3.9

For amino acids labeled with * we observe two resonances due to a mixture of E- and Z-isomers of Pro 361 [4]. #: The CSP cannot be determined either because of line-broadening or because the apo-peak has a Euclidian distance larger than ca. 30 Hz (see legend to this table).

Tab. S4: Additional acquisition parameters for 2D NMR experiments

TD denotes the number of increments in the respective dimensions, O1 is the center of the spectral window, SW is the sweep width, AQ is the acquisition time and NS is the number of scans.

Experiment	Field strength	F2 (TD, O1, SW, AQ)	F1 (TD, O1, SW, AQ)	NS
$^{1}H,^{15}N$ TROSY HSQC	500 MHz	2048<br>4.7 ppm<br>16 ppm<br>128 ms	256<br>117.5 ppm<br>35 ppm<br>72 ms	32
$^{1}H,^{13}C$ HMQC<br>(Methyl TROSY)	600 MHz	512<br>0.8 ppm<br>3.5 ppm<br>122 ms	256<br>17 ppm<br>18 ppm<br>47 ms	8

Tab. S5: Final concentrations of precursors for MILVA-labeling of MNV-P-domains.

Amino acid	Precursor	Concentration
L^{ProS} , V^{ProS}	2-^{13}C-methyl-4-d_3-acetolactate	195 mg/L
I	2-ketobutyricacid-4-^{13}C-3, 3-d_2	72 mg/L
A	L-alanine-^{13}C-d_2<br>succinate-d_4	0.6 g/L<br>3.75 g/L
M	L-methionine-(methyl-^{13}C)	130 mg/L

Table S6: PRODIGY server predictions for monomer-monomer interaction energies in P-domain dimers

Interaction analysis for HuNoV P-domain GII.4 Saga (PDB 4oox) using the PRODIGY[11,12] server

No. of intermolecular contacts	160
No. of charged-charged contacts	9
No. of charged-polar contacts	12
No. of charged-apolar contacts	34
No. of polar-polar contacts	7
No. of apolar-polar contacts	62
No. of apolar-apolar contacts	36
Percentage of apolar NIS* residues	38.35
Percentage of charged NIS* residues	19.90
Predicted binding affinity (kcal mol^{-1})	-23.0
Predicted dissociation constant (M) at 25.0˚C	1.4×10^{-17}

* NIS - Non-Interacting Surfaces

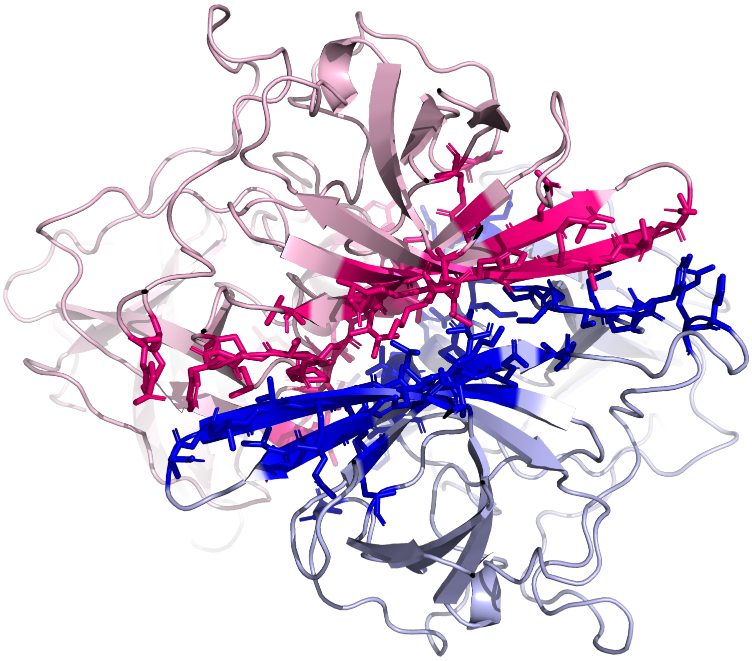

Interaction analysis for MNV CW1 P-domain (PDB 3lq6) using the PRODIGY server[11,12]

No. of intermolecular contacts	138
No. of charged-charged contacts	9
No. of charged-polar contacts	15
No. of charged-apolar contacts	37
No. of polar-polar contacts	5
No. of apolar-polar contacts	49
No. of apolar-apolar contacts	23
Percentage of apolar NIS* residues	47.13
Percentage of charged NIS* residues	21.61
Predicted binding affinity (kcal mol^{-1})	-18.8
Predicted dissociation constant (M) at 25.0°C	1.5 x 10^{-14}

* NIS - Non-Interacting Surfaces

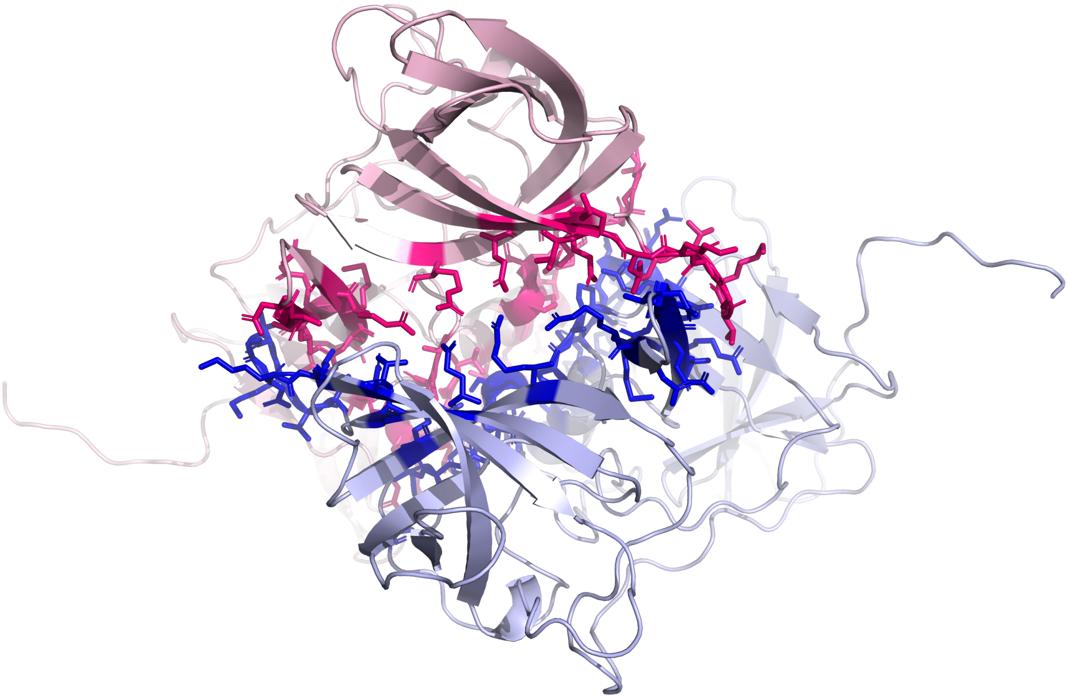

Supplementary Notes

Supplementary Note 1: Shell scripts for NMR data processing with NMRPipe

Processing of concentration dependent methyl TROSY spectra of MNV CW1 P-domain

```
#!/bin/csh

foreach spec (2 4 6 8 10 12)

cd $spec

bruk2pipe -in ./ser \
  -bad 0.0 -ext -aswap -AMX -decim 9520 -dspfvs 20 -grpdly 67.9847717285156  \
  -xN              512  -yN              256  \
  -xT              256  -yT              128  \
  -xMODE           DQD  -yMODE        States-TPPI  \
  -xSW        2100.840  -ySW        2717.391  \
  -xOBS        600.230  -yOBS        150.931  \
  -xCAR          0.800  -yCAR         19.76  \
  -xLAB             1H  -yLAB           13C  \
  -ndim              2  -aq2D     States-TPPI  \
  -out ./test.fid -verb -ov

nmrPipe -in test.fid \
#| nmrPipe -fn SOL                              \
| nmrPipe -fn EM  -lb 4.0 -c 0.5                  \
| nmrPipe  -fn ZF -auto                        \
| nmrPipe  -fn FT -auto                        \
| nmrPipe  -fn PS -p0 58.00 -p1 0.00 -di -verb        \
#| nmrPipe -fn EXT -x1 6ppm -xn 12ppm -sw                          \
| nmrPipe  -fn TP                            \
| nmrPipe -fn LP -fb                              \
| nmrPipe -fn EM  -lb 8.0 -c 0.5                  \
| nmrPipe  -fn ZF -auto                        \
| nmrPipe  -fn FT -auto                        \
| nmrPipe  -fn PS -p0 0 -p1 0 -di -verb        \
   -ov -out ../test-$spec.ft2

rm test.fid

cd ..

end
```

Processing of methyl TROSY spectra of MNV CW1 P-domain titrated with GCDCA

```
#!/bin/csh

foreach spec (200 4 6 8 10 1200 14)

cd $spec

bruk2pipe -in ./ser \
  -bad 0.0 -ext -aswap -AMX -decim 9520 -dspfvs 20 -grpdly 67.9847717285156  \
  -xN              512  -yN              256  \
  -xT              256  -yT              128  \
  -xMODE           DQD  -yMODE        States-TPPI  \
  -xSW        2100.840  -ySW        2717.391  \
  -xOBS        600.230  -yOBS        150.931  \
  -xCAR          0.800  -yCAR         19.76  \
  -xLAB             1H  -yLAB           13C  \
  -ndim              2  -aq2D    States-TPPI  \
  -out ./test.fid -verb -ov

nmrPipe -in test.fid \
#| nmrPipe -fn SOL                              \
| nmrPipe -fn EM  -lb 4.0 -c 0.5                 \
| nmrPipe  -fn ZF -auto                      \
| nmrPipe  -fn FT -auto                      \
| nmrPipe  -fn PS -p0 58.00 -p1 14.00 -di -verb        \
#| nmrPipe -fn EXT -x1 6ppm -xn 12ppm -sw                        \
| nmrPipe  -fn TP                          \
| nmrPipe -fn LP -fb                           \
| nmrPipe -fn EM  -lb 8.0 -c 0.5                 \
| nmrPipe  -fn ZF -auto                      \
| nmrPipe  -fn FT -auto                      \
| nmrPipe  -fn PS -p0 -17.00 -p1 36.00 -di -verb        \
   -ov -out ../test-$spec.ft2

rm test.fid

cd ..

end
```

Supplementary References

1 McWilliam, H. *et al.* Analysis Tool Web Services from the EMBL-EBI. *Nucleic Acids Res* **41**, W597-600, doi:10.1093/nar/gkt376 (2013).

2 Sievers, F. *et al.* Fast, scalable generation of high-quality protein multiple sequence alignments using Clustal Omega. *Mol Syst Biol* **7**, 539, doi:10.1038/msb.2011.75 (2011).

3 Goujon, M. *et al.* A new bioinformatics analysis tools framework at EMBL-EBI. *Nucleic Acids Res* **38**, W695-699, doi:10.1093/nar/gkq313 (2010).

4 Maass, T. *et al.* Assignment of Ala, Ile, LeuproS, Met, and ValproS methyl groups of the protruding domain of murine norovirus capsid protein VP1 using methyl–methyl NOEs, site directed mutagenesis, and pseudocontact shifts. *Biomolecular NMR assignments*, doi:10.1007/s12104-022-10066-7 (2022).

5 Waudby, C. A., Ramos, A., Cabrita, L. D. & Christodoulou, J. Two-Dimensional NMR Lineshape Analysis. *Scientific Reports* **6**, 24826, doi:10.1038/srep24826 (2016).

6 Lakomek, N. A., Ying, J. & Bax, A. Measurement of 15N relaxation rates in perdeuterated proteins by TROSY-based methods. *J Biomol NMR* **53**, 209-221, doi:10.1007/s10858-012-9626-5 (2012).

7 Mallagaray, A. *et al.* A post-translational modification of human Norovirus capsid protein attenuates glycan binding. *Nat Commun* **10**, 1320, doi:10.1038/s41467-019-09251-5 (2019).

8 Kolawole, A. O. *et al.* Newly isolated mAbs broaden the neutralizing epitope in murine norovirus. *J Gen Virol* **95**, 1958-1968, doi:10.1099/vir.0.066753-0 (2014).

9 Nelson, C. A. *et al.* Structural basis for murine norovirus engagement of bile acids and the CD300lf receptor. *Proc Natl Acad Sci U S A* **115**, E9201-E9210, doi:10.1073/pnas.1805797115 (2018).

10 Creutznacher, R. *et al.* Chemical-Shift Perturbations Reflect Bile Acid Binding to Norovirus Coat Protein: Recognition Comes in Different Flavors. *Chembiochem* **21**, 1007-1021, doi:10.1002/cbic.201900572 (2020).

11 Xue, L. C., Rodrigues, J. P., Kastritis, P. L., Bonvin, A. M. & Vangone, A. PRODIGY: a web server for predicting the binding affinity of protein-protein complexes. *Bioinformatics* **32**, 3676-3678, doi:10.1093/bioinformatics/btw514 (2016).

12 Vangone, A. & Bonvin, A. M. Contacts-based prediction of binding affinity in protein-protein complexes. *Elife* **4**, e07454, doi:10.7554/eLife.07454 (2015).
