# Supplementary material for: Distinct dissociation rates of murine and human norovirus P-domain dimers suggest a role of dimer stability in virus-host interactions
Source: Commun Biol. 2022 Jun 9;5:563. doi: 10.1038/s42003-022-03497-4 (PMC9184547; doi:10.1038/s42003-022-03497-4)
Supplement: Supplementary file 3 — Description of Additional Supplementary Files [file 42003_2022_3497_MOESM3_ESM.pdf]

Description of Additional Supplementary Files

File name: Supplementary Data 1
Description: Excel file (.xlsx) containing the source data behind the graphs in Figs. 1c, 2a, 2b, 3a, and 3b, and in the supplementary figures Figs. S4, S5, S6a, S6b, S11, S12, S13b, S13c, S16, S17, S19, and S20.

File name: Supplementary Data 2
Description: Zip-file (.zip) containing the NMR raw data for the concentration dependent methyl TROSY spectra (Fig. S9). Data sets 2, 4, 6, 8, and 12 contain the 2D series files (.ser) for MNV CW1 P-domain concentrations of 13, 25, 50, 75, 100, and 230 µM, respectively. The data sets 1, 3, 5, 7, and 11 contain 1D 1H NMR fids (.fid) for quality control.

File name: Supplementary Data 3
Description: Zip-file (.zip) containing the NMR raw data for the titration of MNV CW1 P-domain with GCDCA (Fig. 5 and Fig. S22). Data sets 2, 4, 6, 8, 12, and 14 contain the 2D series files (.ser) for a sample of MNV CW1 P-domain (50 µM) Page 7 of 15 in the presence of GCDCA concentrations of 0, 25, 50, 75, 125, 250, and 550 µM, respectively. The data sets 1, 3, 5, 7, 11, and 13 contain 1D 1H NMR fids (.fid) for quality control.
