# Supplementary material for: Distinct dissociation rates of murine and human norovirus P-domain dimers suggest a role of dimer stability in virus-host interactions
Source: Commun Biol. 2022 Jun 9;5:563. doi: 10.1038/s42003-022-03497-4 (PMC9184547; doi:10.1038/s42003-022-03497-4)
Supplement: Supplementary file 7 — Reporting Summary [file 42003_2022_3497_MOESM7_ESM.pdf]

nature research

Corresponding author(s): Thomas Peters and Stefan Taube

Last updated by author(s): Mar 3, 2022

Reporting Summary

Nature Research wishes to improve the reproducibility of the work that we publish. This form provides structure for consistency and transparency in reporting. For further information on Nature Research policies, see our Editorial Policies and the Editorial Policy Checklist.

Statistics

For all statistical analyses, confirm that the following items are present in the figure legend, table legend, main text, or Methods section.

n/a	Confirmed	
☐	☒	The exact sample size (*n*) for each experimental group/condition, given as a discrete number and unit of measurement
☐	☒	A statement on whether measurements were taken from distinct samples or whether the same sample was measured repeatedly
☐	☒	The statistical test(s) used AND whether they are one- or two-sided<br>*Only common tests should be described solely by name; describe more complex techniques in the Methods section.*
☐	☒	A description of all covariates tested
☐	☒	A description of any assumptions or corrections, such as tests of normality and adjustment for multiple comparisons
☐	☒	A full description of the statistical parameters including central tendency (e.g. means) or other basic estimates (e.g. regression coefficient) AND variation (e.g. standard deviation) or associated estimates of uncertainty (e.g. confidence intervals)
☐	☒	For null hypothesis testing, the test statistic (e.g. *F*, *t*, *r*) with confidence intervals, effect sizes, degrees of freedom and *P* value noted<br>*Give P values as exact values whenever suitable.*
☐	☒	For Bayesian analysis, information on the choice of priors and Markov chain Monte Carlo settings
☐	☒	For hierarchical and complex designs, identification of the appropriate level for tests and full reporting of outcomes
☐	☒	Estimates of effect sizes (e.g. Cohen's *d*, Pearson's *r*), indicating how they were calculated

Our web collection on statistics for biologists contains articles on many of the points above.

Software and code

Policy information about availability of computer code

Data collection	NMR data acquisition: TopSpin 3.6 (Bruker)
Data analysis	NMR data analysis: TopSpin 3.6, CCPNMR 2.4.2, TITAN Matlab scripts, Matlab and Python scripts; Ion exchange chromatography data analysis: Python scripts; MS data analysis: MassLynx, Origin Pro 2016, Excel

For manuscripts utilizing custom algorithms or software that are central to the research but not yet described in published literature, software must be made available to editors and reviewers. We strongly encourage code deposition in a community repository (e.g. GitHub). See the Nature Research guidelines for submitting code & software for further information.

Data

Policy information about availability of data

All manuscripts must include a data availability statement. This statement should provide the following information, where applicable:

- Accession codes, unique identifiers, or web links for publicly available datasets
- A list of figures that have associated raw data
- A description of any restrictions on data availability

NMR 13C-methyl group chemical shift assignments for GCDCA-bound MNV CW1 P-dimers have been published separately (Maass, T., Westermann, L.T., Creutznacher, R., Mallagaray, A., Dülfer, J., Uetrecht, C., and Peters, T. (2022). Assignment of Ala, Ile, LeuproS, Met, and ValproS methyl groups of the protruding domain of murine norovirus capsid protein VP1 using methyl–methyl NOEs, site directed mutagenesis, and pseudocontact shifts. Biomolecular NMR assignments. DOI 10.1007/s12104-022-10066-7). The assignments are deposited with the BioMagResBank ((https:// www. bmrb. wisc. edu) under the accession number 50919. The transfer of assignments to the apo-form is compiled in Tab. S3. Figure 1 contains raw NMR and MS data (spectra) and SEC (size exclusion chromatography) data (normalized). Figure 2 contains ion exchange chromatograms (IEX) and raw MS data. Figure 3 contains SEC data and raw data from differential scanning fluorimetry

(DSF). Figure 4 contains raw NMR data. Figure 5 contains no raw data. There are no restrictions on data availability. All data are available from the authors upon request.

Field-specific reporting

Please select the one below that is the best fit for your research. If you are not sure, read the appropriate sections before making your selection.

☒ Life sciences ☐ Behavioural & social sciences ☐ Ecological, evolutionary & environmental sciences

For a reference copy of the document with all sections, see nature.com/documents/nr-reporting-summary-flat.pdf

Life sciences study design

All studies must disclose on these points even when the disclosure is negative.

Sample size	Side chain methyl isotope (MILVA) labeled protein samples were subjected to heteronuclear chemical shift perturbation (CSP) NMR experiments. CSP NMR experiments are commonly based on a single data set. For the analysis of dissociation constants from CSP NMR experiments and for the analysis of exchange kinetics based on methyl TROSY experiments titration series have been performed. Dissociation constants from MS rely on native MS spectra that for each protein concentration, i.e., 4 measurements for CR10 and 3 measurements for MNV07. Thermal stability assays and plaque assays have been performed in triplicate.
Data exclusions	No data were excluded from analysis.
Replication	NMR spectra of different batches of protein preparations were identical, i.e., chemical shifts are identical. Other experimental data were obtained at least in triplicate.
Randomization	The experimental setup is such that randomization is not required.
Blinding	Blinding is not applicable in our studies.

Reporting for specific materials, systems and methods

We require information from authors about some types of materials, experimental systems and methods used in many studies. Here, indicate whether each material, system or method listed is relevant to your study. If you are not sure if a list item applies to your research, read the appropriate section before selecting a response.

Materials & experimental systems

n/a	Involved in the study	
☐	☒	Antibodies
☐	☒	Eukaryotic cell lines
☒	☐	Palaeontology and archaeology
☒	☐	Animals and other organisms
☒	☐	Human research participants
☒	☐	Clinical data
☒	☐	Dual use research of concern

Methods

n/a	Involved in the study	
☒	☐	ChIP-seq
☒	☐	Flow cytometry
☒	☐	MRI-based neuroimaging

Antibodies

Antibodies used	2D3, 4F9 and A6.2 from Prof. Wobus laboratory (University of Michigan, USA)
Validation	N.A.

Eukaryotic cell lines

Policy information about cell lines

Cell line source(s)	*State the source of each cell line used.*
Authentication	Procedure for authentication: ELISA
Mycoplasma contamination	Confirmed
Commonly misidentified lines (See ICLAC register)	N.A.
